# Characteristics of medicine use for children with asthma in China: a nationwide population-based study

**DOI:** 10.1186/s12887-022-03720-5

**Published:** 2022-12-28

**Authors:** Ping Wu, Baoping Xu, Adong Shen, Huasong Zeng, Kunling Shen

**Affiliations:** 1 Department of Allergy, Immunology and Rheumatology, Guangzhou Women and Children’s Medical Center, Guangzhou Medical University, No. 318 Renmin Middle Road, Yuexiu District, Guangzhou, 510120 Guangdong China; 2 China National Clinical Research Center of Respiratory Diseases, Respiratory Department of Beijing Children’s Hospital, Capital Medical University, National Center for Children’s Health, No. 56 Nan Li Shi Road, Xicheng District, Beijing, 100045 China

**Keywords:** Pediatrics, Asthma, Medication, Healthcare insurance data

## Abstract

**Objective:**

To analyze the asthma medication use in Chinese children of different age groups, regions, and levels of cities in China, based on the 2015 Healthcare Insurance Data in China.

**Methods:**

The China Healthcare Insurance Research Association (CHIRA) database was searched for children from 0 to 14 years old diagnosed as asthma based on the “J45” and “J46” coded in ICD-10. A cross-sectional study design was employed.

**Results:**

A total of 308,550 children were identified, all of whom were treated under the coverage of healthcare insurance. Among them, 2,468 children were eligible for inclusion in the present study. Compared with the current status of asthma care in European and American countries, under the guidelines for the diagnosis and treatment of asthma in China, the use percentages of ICS and short-acting β_2_ receptor agonist in children with asthma in China were lower, but the use percentages of oral corticosteroids, long-acting β_2_ receptor agonist, and theophylline (especially intravenous theophylline) were higher, especially in the Central and West China.

**Conclusion:**

The asthma medication use was attributed to many factors, thus efforts are still needed to further popularize the GINA programs and China's guidelines for asthma diagnosis and treatment, especially in the Central and West China.

**Supplementary Information:**

The online version contains supplementary material available at 10.1186/s12887-022-03720-5.

## Introduction

It is estimated that there are at least 300 million asthma patients in the world, with mainland China accounting for about 10%, approximately 30 million [1, 2]. In 2010, the cumulative prevalence of asthma reached 3.02% in urban children (0–14 years) nationwide [3, 4]. According to the Global Initiative for Asthma (GINA) program, the goal of asthma management in clinics is to achieve and maintain control of asthma symptoms over a long period of time. Complete control of asthma relies on effective use of evidence-based medications to treat asthma, and the efficacy of these drugs has been outlined in the asthma guidelines [5].

There are remarkable differences in the social and economic development of various regions in China. Therefore, the choice of asthma treatment approaches may be related not only to the efficacy of clinically available drugs and age of the patients, but also to regional, economic and cultural differences in the various regions. According to the third national urban asthma survey data of children aged from 0 to 14 years old reported in 2013, among the 13,992 children with asthma, the proportion of bronchodilator users was 71.4%, and the proportion of inhaled corticosteroids users was 58.7% [6]. In recent years, there have been few studies on the characteristics of anti-asthma medication among children in China. China’s Healthcare Insurance Research Association (CHIRA) database provides us with clinical data on pediatric patients with asthma who are under the national health insurance coverage. The present study was designed to investigate the medication usage status of children with asthma in different age groups, different regions and different levels of cities in China, and to compare them with international and domestic guidelines, with a purpose of providing a basis for standardized usage of medication for children with asthma in China.

## Materials and methods

**Fig. 1:**
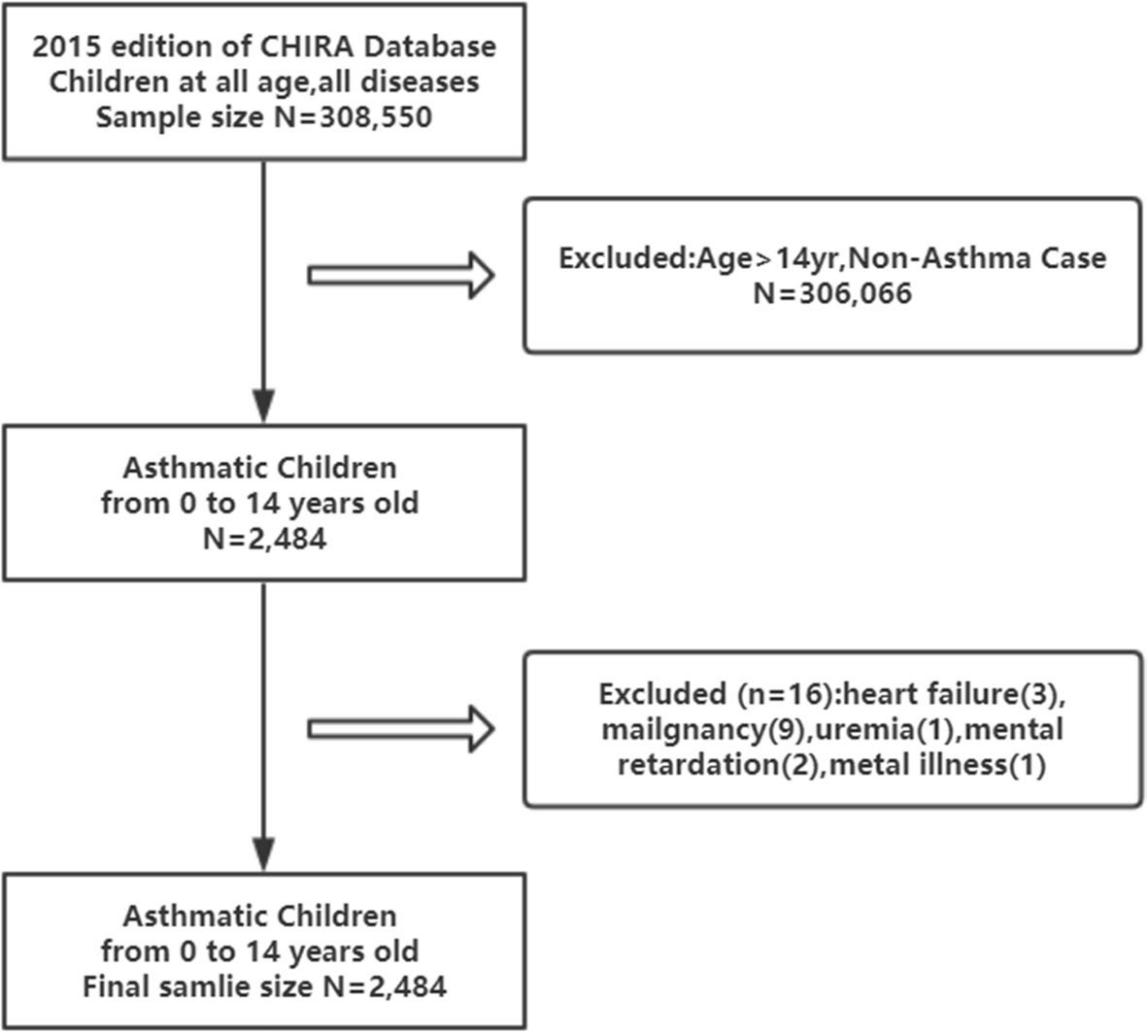
The selection process of the children included in the analysis. Children from 0 to 14 years old were included, who had a diagnosis of asthma, identified by the International Statistical Classification of Diseases and Related Health Problems revision 10 (ICD-10) code (ICD-10 J45 and J46). Various clinical types of asthma was included, namely bronchial asthma, cough variant asthma, and infantile asthma, but those children diagnosed as asthmatic bronchitis were excluded. Considering that several severe and chronic diseases or conditions might affect the medication for children with asthma, patients with heart failure, malignancy, uremia, intellectual disability, or mental illness were excluded from the present study

Details are shown in the separate article that we have published. Please refer to reference No. 7. A total of 308,550 children were identified, all of whom were treated under the coverage of healthcare insurance. Among them, 2,468 children were eligible for inclusion in the present study. The selection process was shown in Fig. 1.

### Data sources

The 2015 edition of CHIRA database was searched. The CHIRA database is an administrative healthcare insurance management information database initiated in 2007 and managed by the CHIRA. The samples covered 46 cities across the country, including 19 in the East China, 15 in the Central China and 12 in the West China [7].

This project is a subtopic of a National Soft Science Item, termed “Investigation on the Utilization of Drugs, Medical Devices and Medical Diagnosis and Treatment Projects in Insured Patients with National Urban Basic Healthcare Insurance Coverage”, of the Ministry of Human Resources and Social Security in China. The determination of sampling proportion was mainly based on several factors: first, data extraction pressure, to guarantee the data extraction did not affect the normal operation of the system; second, data security, to accommodate research needs with low morbidity as far as possible; third, the ability to clean up and standardize data. The proportion of 2% of capital cities and municipalities and 5% of prefecture-level cities was thus determined.

### Outcome measures

Patient information identified from the CHIRA database included age, sex, comorbidities, and healthcare insurance type (Urban Employee Basic Healthcare Insurance or Urban Residents Basic Healthcare Insurance). Medications prescribed in 2015 for the treatment of childhood asthma were identified and categorized according to the class of medication including asthma control drugs and asthma relief drugs (details are presented in the Supplementary Table 1). Chinese patent medicines are manufactured products with a standardized composition of herbal extracts and other ingredients, generally available as pills, capsules, or liquids. Healthcare resource utilization included the number of visits to different levels of hospitals, the hospital level and department of the patient’s first asthma-related visit in 2015.

Subgroup analyses were conducted based on different age groups, locations, and city level. As for the age, children were further classified as group A, infants and young children (0–2 years old), group B, preschool children (3–6 years old), and group C, school-age children (7–14 years old). Totaling three regions of China were classified, according to the geographical characteristics, namely East China, Central China, and West China. And 5 levels of city grade were noted, viz. cities of 1^st^, 2^nd^, 3^rd^, as well as 4^th^ tiers and below.

The East China includes ten provinces and municipalities, viz. Beijing, Tianjin, Hebei, Liaoning, Jiangsu, Zhejiang, Fujian, Shandong, Guangdong and Hainan. The Central China includes eight provinces, viz. Jilin, Heilongjiang, Shanxi, Anhui, Jiangxi, Henan, Hubei and Hunan. The West China includes eight provinces, autonomous regions and municipalities directly under the central government, namely Inner Mongolia, Guangxi, Chongqing, Sichuan, Guizhou, Yunnan, Shanxi and Qinghai.

According to the China Yearbooks in 2015, all cities were divided into different levels according to their comprehensive level that including the political, economic and cultural level, medical resources level, and so on. The 1^st^ tier cities refer to metropolises, including Beijing and Guangzhou that play an important role in the national political, economic and other social activities and have a leading role and the ability to radiate and drive them. The 2^nd^ tier cities include Hangzhou, Jinan, Shenzhen, Tianjin, Chengdu, Chongqing and Wuhan. The 3^rd^ tier cities refer to medium-sized cities with strategic significance or relatively developed or large economic aggregate. Most of them are cities in the Central China, prefecture-level cities with more developed economic conditions and national top 100 counties in the Central and East China. It also includes the capital cities of some provincial capitals in the West China, such as Dalian, Dongguan, Fuzhou, Xiamen, Zibo, Nantong, Shijiazhuang, Guiyang, Kunming, Xi'an, Zunyi, Changchun, Harbin, Nanchang, Changsha, Taiyuan, Zhengzhou. The 4^th^ tiers and below cities include all county-level cities and counties yet excluding those in the 1^st^, 2^nd^, and 3^rd^, including Sanya, Haikou, Jinhua, Lianyungang, Qinhuangdao, Weifang, Baotou, Haibei Tibetan autonomous prefecture, Liuzhou, Mianyang, Xianyang, Yuxi, Anqing, Datong, Jingzhou, Jiujiang, Luoyang, Tonghua, Xiangyang, Yueyang.

### Statistical analysis

Using cross-sectional descriptive statistical analysis methods, categorical variables (e.g., gender) were expressed as percentages, and continuous numerical variables (e.g., age) were expressed as means with standard deviations. Chi-square test was used for comparison between groups. *P* < 0.05 is considered statistically significant. Statistical software used in the present study includes SAS 9.2, Access, and Microsoft Excel.

## Results

### Demographic characteristics of the patients

The demographic characteristics was shown in Table 1. The average age of children with asthma was 5.50 ± 3.32 years old, where males accounted for 63.82%, and females accounted for 36.18%[7]. There’s no statistical difference among different groups.

**Table 1:** Demographic characteristics

**Items**	**Total** **(*n* = 2468)**	**Group A** **(*n* = 439)**	**Group B** **(*n* = 967)**	**Group C** **(*n* = 1062)**	***P***
Age (years), mean ± SD	5.50 ± 3.32	1.36 ± 0.34	3.92 ± 0.80	8.66 ± 2.35	
Sex, n (%)					
Male	1575 (63.82%)	300 (68.34%)	594 (61.43%)	681 (64.12%)	.04
Female	893 (36.18%)	139 (31.66%)	373 (38.57%)	381 (35.88%)	
Clinical visits, n (%)					
Outpatient	70,570 (98.58%)	14,419 (98.11%)	32,722 (98.76%)	23,429 (98.63%)	< .01
Inpatient	1014 (1.42%)	278 (1.89%)	411 (1.24%)	325 (1.37%)	
Hospital grade, n (%)					
Tertiary	44,440 (62.08%)	9304 (63.31%)	20,325 (61.34%)	14,811 (62.35%)	< .01
Secondary	13,686 (19.12%)	2582 (17.57%)	6808 (20.55%)	4296 (18.09%)	
Primary	13,433 (18.77%)	2811 (19.13%)	5994 (18.09%)	4628 (19.48%)	
Hospital type, n (%)					
General	51,731 (72.27%)	8554 (58.20%)	24,147 (72.88%)	19,030 (80.11%)	< .01
Specialized	19,828 (27.70%)	6143 (41.80%)	8980 (27.10%)	4705 (19.81%)	
Regions, n (%)					
East China	1900(76.99%)	352(80.18%)	756(78.18%)	792(74.58%)	< .01
Central China	223(9.04%)	44(10.02%)	97(10.03%)	82(7.72%)	
West China	345(13.98%)	43(9.79%)	114(11.79%)	188(17.70%)	
City Levels, n(%)					
1^st^-tier	601(24.35%)	74(16.86%)	246(25.44%)	281(26.46%)	< .01
2^nd^-tier	1268(51.38%)	289(65.83%)	522(53.98%)	457(43.03%)	
3^rd^-tier	195(7.90%)	29(6.61%)	77(7.96%)	89(8.38%)	
4^th^ and 5^th^ tier	314(12.72%)	47(10.71%)	122(12.62%)	145(13.65%)	

### The status of medications used for asthma control among the different age groups, regions, and levels of cities

**Fig. 2:**
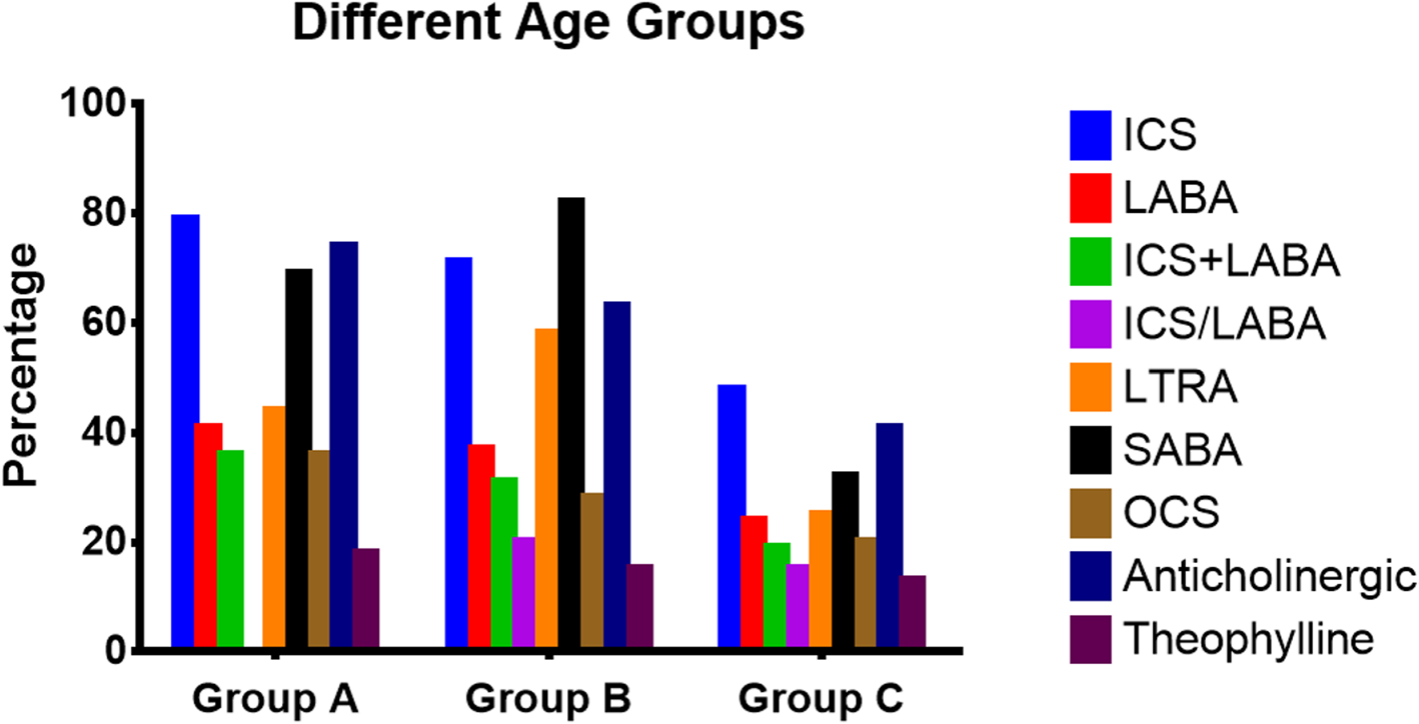
The status of asthma control and relief drug usage among children in the different age groups

**Fig. 3:**
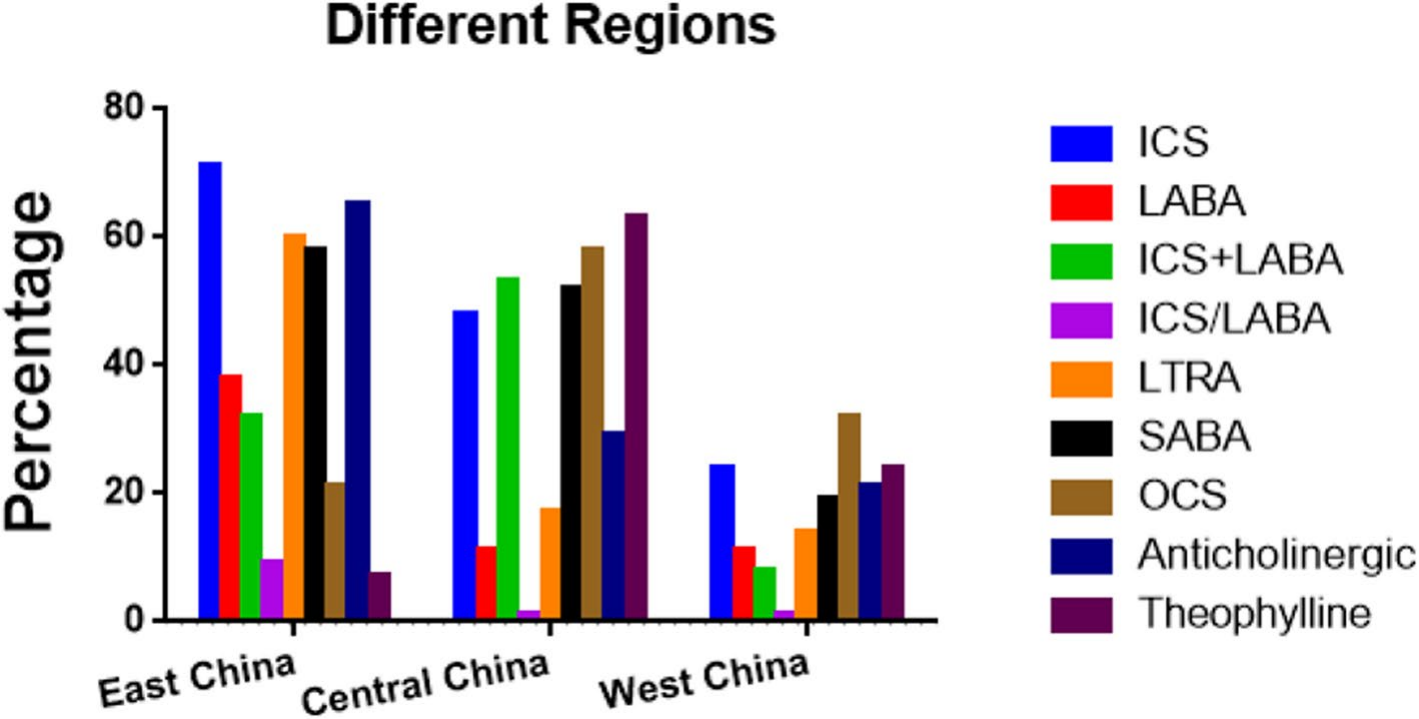
The status of asthma control and relief drug usage among children in the different regions

**Fig. 4:**
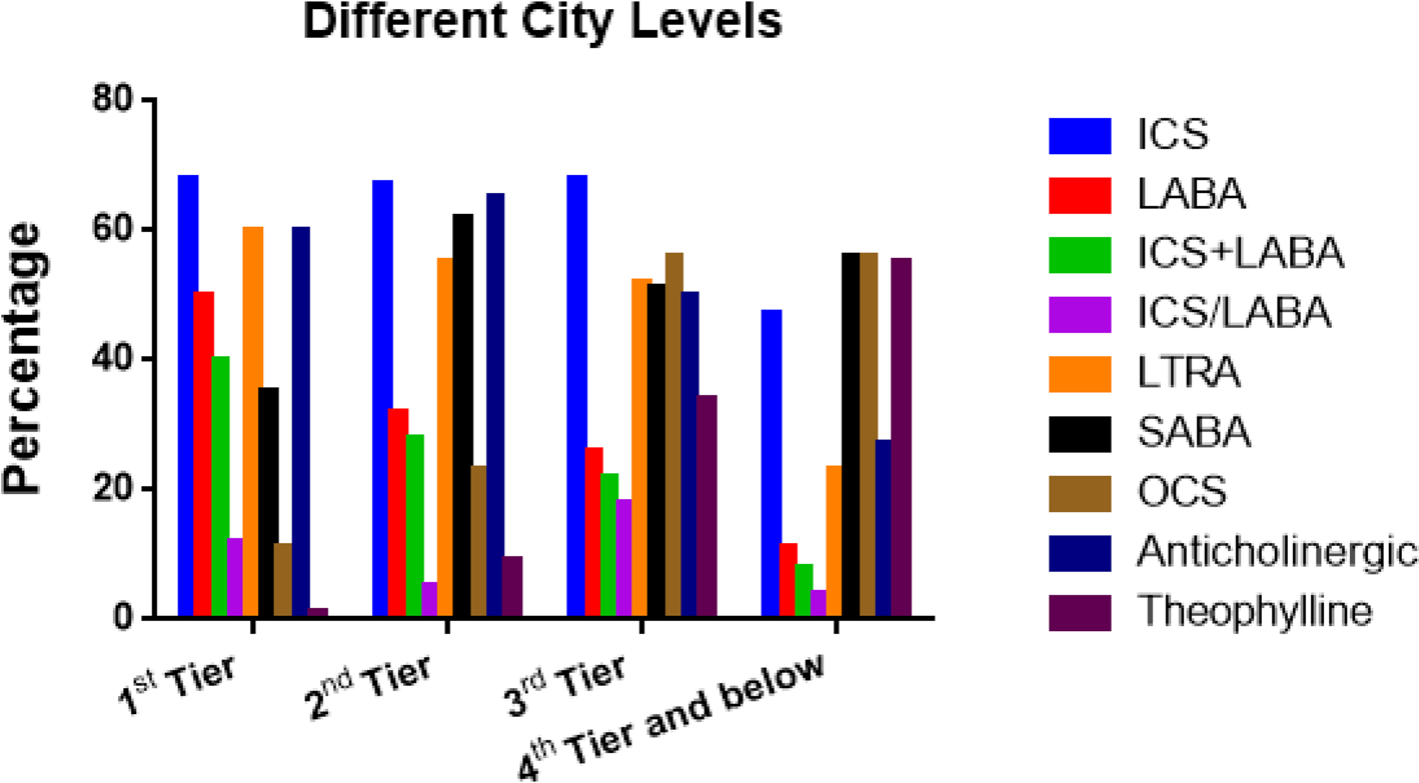
The status of asthma control and relief drug usage among children in the different city levels

As shown in Table 2 and Figs. 2, 3 and 4, there were significant differences in the status of asthma control associated with the drug usage among children in different age groups, regions and levels of urban cities.

**Table 2:** The status of asthma control drug usage among children in the different age groups, regions and city levels

	**Age groups**				**Regions**				**City levels**				
	**Children younger than 3 years old**	**Preschool children**	**School-age children**	*P*	**East China**	**Central China**	**West China**	***P***	**1**^**st**^** Tier**	**2**^**nd**^** Tier**	**3**^**rd**^** Tier**	**4**^**th**^** and 5**^**th**^** Tier**	***P***
	**(*N* = 439)**	**(*N* = 967)**	**(*N* = 1062)**		**(*N* = 1900)**	**(*N* = 223)**	**(*N* = 345)**		**(*N* = 601)**	**(*N* = 1268)**	**(*N* = 195)**	**(*N* = 314)**	
**ICS**	346 (78.82%)	687 (71.04%)	512 (48.21%)	< .0001	1356(71.37%)	106(47.53%)	83(24.06%)	< .0001	411(68.39%)	854(67.35%)	132(67.69%)	148(47.13%)	< .0001
**LABA**	181 (41.23%)	354 (36.61%)	258 (24.29%)	< .0001	731(38.47%)	25(11.21%)	37(10.72%)	< .0001	301(50.08%)	405(31.94%)	51(26.15%)	36(11.46%)	< .0001
**ICS + LABA**^**a**^	160 (36.45%)	300 (31.02%)	198 (18.64%)	< .0001	613(32.26%)	19(53%)	26(7.54%)	< .0001	239(39.77%)	350(27.60%)	43(22.05%)	26(8.28%)	< .0001
**ICS/LABA**^**b**^	0	195 (20.17%)	158 (14.88%)	< .0001	180(9.47%)	3(1.35%)	5(1.45%)	< .0001	75(12.48%)	66(5.21%)	36(18.46%)	11(3.50%)	< .0001
**LTRA**	195(44.42%)	558 (57.70%)	270 (25.42%)	< .0001	1141(60.05%)	37(16.59%)	47(13.62%)	< .0001	359(59.73%)	693(54.65%)	101(51.79%)	72(22.93%)	< .0001

^**a**^ICS + LABA means both of ICS and LABA are used together

^**b**^LABA/ICS is a compound preparation

The overall use percentages of inhaled corticosteroids (ICS) was 62.60% (1545/2468), and there were significant differences in the percentages among different age groups, regions and levels of cities (*P* < 0.05). The ICS use percentages were the lowest in school-age children, West China, and the 4^th^ tier and below cities, with the percentages being 48.21% (512/1062), 24.06% (83/345), and 47.13% (148/314), respectively.

The administration method of ICS in children younger than 3 years old and pre-school children is mainly nebulized inhalation (100%), while in school-age children is including nebulized inhalation (273/512, 53.32%), aerosol inhalation (209/512,40.82%) and powder inhalation(30/512, 5.86%). Details are shown in Table 3.

**Table 3:** The administration method of ICS in the different age groups

	**Age groups**			
	**Children younger than 3 years old**	**Preschool children**	**School-age children**	*P*
	**(*N* = 439)**	**(*N* = 967)**	**(*N* = 1062)**	
**ICS**	346 (78.82%)	687 (71.04%)	512 (48.21%)	< .0001
**Nebulized Inhalation**	346(100%)	684(100%)	273/512(53.32%)	< .0001
**Aerosol Inhalation**	-	-	209/512(40.82%)	-
**Powder Inhalation**	-	-	30/512(5.86%)	-

The overall use percentage of long-acting β_2_ receptor agonist (LABA) was 32.13% (793/2468), and there were significant differences in the rate among different age groups, regions and levels of cities (*P* < 0.05). The usage percentages of LABA alone were the highest in children younger than 3 years old, East China and the 1^st^ tier cities, with the percentages being 41.23% (181/439), 38.47% (731/1900) and 50.08% (301/411) respectively.

### The status of asthma relief drug usage among children in different age groups, regions and levels of cities

**Fig. 5:**
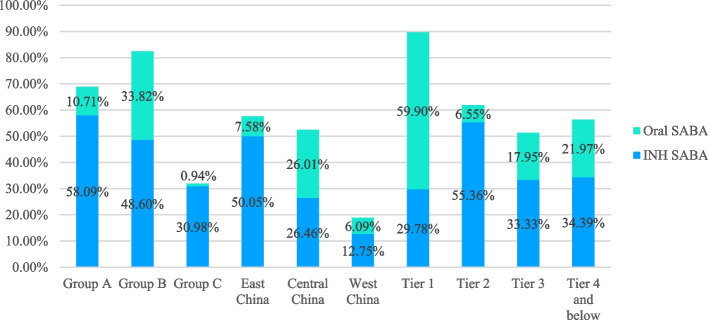
The status of oral and inhaled SABA in each subgroup

**Fig. 6:**
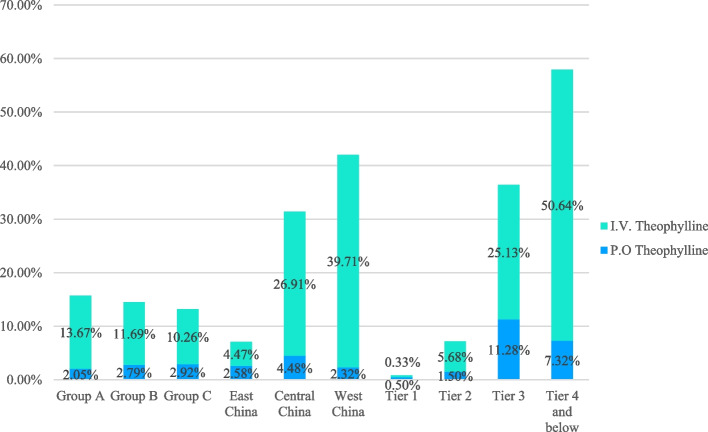
The status of oral and intravenous theophylline proportions in each subgroup

As shown in Tables 4 and 5 and Figs. 2, 3, 4, 5 and 6, the overall use percentage of short-acting β_2_ receptor agonist (SABA, mainly by inhalation) was 51.74% (1277/2468), and there were significant differences in the percentage among different age groups, regions and levels of cities (*P* < 0.05). The SABA use percentage was the lowest in school-age children, West China and 1^st^ tier level cities, which were 31.92% (339/1062), 18.84% (65/345), and 35.77% (215/601), respectively.

**Table 4:** The status of asthma relief drug usage among children in different age groups, regions and city levels

	**Age groups**				**Regions**				**City levels**				
	**Children younger than 3 years old**	**Preschool children**	**School-age children**	*P*	**East China**	**Central China**	**West China**	*P*	**1**^**st**^** Tier**	**2**^**nd**^** Tier**	**3**^**rd**^** Tier**	**4**^**th**^** and 5**^**th**^** Tier**	*P*
	**(*N* = 439)**	**(*N* = 967)**	**(*N* = 1062)**		**(*N* = 1900)**	**(*N* = 223)**	**(*N* = 345)**		**(*N* = 601)**	**(*N* = 1268)**	**(*N* = 195)**	**(*N* = 314)**	
**SABA**													
**Inhalation**	255 (58.09%)	470 (48.60%)	329 (30.98%)	< .0001	951(50.05%)	59(26.46%)	44(12.75%)	< .0001	179(29.78%)	702(55.36%)	65(33.33%)	108(34.39%)	< .0001
**Oral**	47 (10.71%)	327 (33.82%)	10 (0.94%)	< .0001	144(7.58%)	58(26.01%)	21(6.09%)	< .0001	36(59.90%)	83(6.55%)	35(17.95%)	69(21.97%)	< .0001
**OCS**	158 (36.00%)	270 (27.92%)	217 (20.43%)	< .0001	406(21.37%)	130(58.30%)	109(31.59%)	< .0001	67(11.15%)	291(22.95%)	110(56.41%)	177(56.37%)	< .0001
**SAAC**	327 (74.49%)	605 (62.56%)	431 (40.58%)	< .0001	1228(64.63%)	64(28.70%)	71(20.58%)	< .0001	361(60.07%)	819(64.59%)	97(49.74%)	86(27.39%)	< .0001
**Theophylline**													
**Oral**	9 (2.05%)	27 (2.79%)	31 (2.92%)	0.63	49(2.58%)	10(4.48%)	8(2.32%)	0.281	3(0.50%)	19(1.50%)	22(11.28%)	23(7.32%)	< .0001
**Intravenous**	60 (13.67%)	113 (11.69%)	109 (10.26%)	0.166	85(4.47%)	60(26.91%)	137(39.71%)	< .0001	2(0.33%)	72(5.68%)	49(25.13%)	159(50.64%)	< .0001

**Table 5:** The use of SAAC in the different hospital subgroups

	**Different hospital types**		**P**	**Different hospital grades**			**P**
	**General hospitals**	**Specialized hospitals**		**Tertiary hospitals**	**Secondary hospitals**	**Primary** **hospitals**	
	**(*N* = 2336)**	**(*N* = 1193)**		**(*N* = 1799)**	**(*N* = 941)**	**(*N* = 1281)**	
**SAAC**	**1115( 47.73%)**	**477 (39.98%)**	** < .0001**	**932 (51.81%)**	**363 (38.58%)**	**337(29.43%)**	** < .0001**

The overall use percentage of oral corticosteroids (OCS) was 26.13% (645/2468). There was significant difference in the percentages among different age groups, regions and levels of cities (*P* < 0.05). The OCS use percentages were the highest in children younger than 3 years old, Central China and 3^rd^ tier cities, with the percentages being 36.00% (158/439), 58.30% (130/223), and 56.41% (110/195) respectively.

The overall use percentage of theophylline was 14.38% (355/2468). There was significant difference in the use percentages among different age groups, regions and levels of cities (*P* < 0.05). The use percentage of intravenous theophylline in 4^th^ tier and below cities was as high as 50.64% (159/314). The use percentages of theophylline were the highest in children younger than 3 years old, Central China and 4^th^ tier and below cities, with the percentages being 17.54% (77/439), 63.23% (141/223), and 55.10% (173/314) respectively.

## Discussion

Previous epidemiological studies on the use of medications to treat asthma were based on the surveys in schools or communities [8, 9], revealing that there was no difference in the estimates of asthma incidence among different age groups and sex, and the general trends of drug use was consistent with the global asthma epidemiology report [10]. The present study was based on health insurance data, which may be more accurate and useful in the analysis of drug use patterns at the national level. The following discussions will focus on the primary medications to treat asthma currently used in clinics.

### Asthma control drugs

#### Inhaled corticosteroids (ICS)

ICS is the preferred preventive treatment for patients with severe and persistent asthma in all age groups, as recommended by national and international guidelines, which are currently regarded as the most effective asthma control drugs [5]. In a cross-sectional study conducted in South Carolina, USA [11], 14,346 (73.5%) of the 19,512 children with asthma aged 2–18 years received ICS from 2007 to 2009.

In 2000, the second epidemiological survey of asthma in urban regions in China showed that the ICS use percentage in children with asthma was 37.13% [12], and the same figure increased to 58.70% in 2010 [6]. In the present study, the overall use percentage of ICS increased to 62.60%, significantly higher than the previous report, with the rates being 48.21% in school-age children, 20.06% in the western region and 47.13% in 4th tier and below cities. Therefore, major efforts should be devoted to educate pediatricians in underdeveloped regions and in urban regions of tier 4 and below in order to enforce asthma treatment guidelines, to improve the awareness of the effectiveness and importance of ICS in treating asthma and to standardize treatment of childhood asthma in those regions.

As is shown in Table 3, nebulized inhalers are mainly used in infants and young children. Aerosol and powder inhalers are mainly used in older children. Those are consistent with the actual clinical situation and conforms to the standards required by domestic and international guidelines.

#### Long-acting β2 receptor agonist (LABA)

LABA is mainly used in combination therapy for asthma in children aged 6 years or older whose symptoms are not fully controlled by moderate dose ICS [5, 13]. The results from a study of Arfã ¨A et al*.* demonstrate that LABA/ICS combination therapy effectively reduces the risk of serious asthma attack [14]. However, Anagnostou et al*.* have shown that LABA may increase the severity of the disease and the risk of asthma-related death in children with severe asthma, especially when LABA is used alone in the absence of conventional treatment with ICS [15]. Some in vitro experiments have shown that repeated bronchoconstriction induces epithelial cell stress, which may eventually lead to remodeling [16]. Bereznicki et al. have reported that 25.7% of children with asthma in Tasmania, Australia, are treated with LABA [17]. In the present study, we found that 32.13% of pediatric patients received LABA monotherapy. It can be concluded that the proportion of LABA monotherapy in China remains very high, therefore Chinese pediatricians need to improve their understanding of the risk of LABA monotherapy and minimize the use of LABA monotherapy in clinics, in order to improve the safety of medication in pediatric patients with asthma.

#### Leukotriene receptor antagonist (LTRA)

Montelukast, a leukotriene modulator, is mainly used as monotherapy in children with mild persistent asthma, especially for children who cannot be treated with ICS or with allergic rhinitis [18]. In a cross-sectional study in South Carolina, USA, 2,508 (12.90%) of the 19,512 children aged 2–18 years with asthma received LTRA monotherapy in 2007–2009 [10]. Bereznicki et al. have reported that 31.3% of children with asthma in Tasmania, Australia, received LTRA treatment in 2011 [17]. In the present study, the use rate of LTRA was 49.64%, significantly higher than that of the third urban asthma epidemiology survey in China in 2010, which was 34.8% [12]. Compared with European and American countries, our LTRA use rate was at a relatively higher level, which may be related to the differences between the guidelines for the diagnosis and treatment of asthma in China and western countries, especially Europe and the United States.

### Asthma relief drugs

#### Inhaled short-acting β2 receptor agonist (SABA)

In the long-term management of pediatric patients with asthma, there is a need for the use of asthma relief drugs according to individual conditions, in addition to the regular and daily use of asthma control drugs. SABA is currently the most effective class of relief drugs, and it is the first choice for the first-line therapy in children with acute asthma in all ages [19]. Children with asthma and/or their parents should be guided to use SABA promptly in the presence of asthma attacks, in accordance with the yellow zone direction of Asthma Action Plan (AAP).

A retrospective study conducted in the United Kingdom in 2011 analyzed the clinical data of 11,641 cases of asthma in children aged 0–18 years from 2001 to 2006, which reported that the SABA prescription rate increased from 88.2% to 91.4% during the 5-year period [20]. Dombkowski et al. have reported that the majority (89.13%) of children with asthma in Michigan, USA, had received at least one SABA prescription [21]. In the present study, the overall use rate of SABA was 51.74% and was predominantly in inhalation form (42.71%). The SABA use rate was 31.92% in school-age children, 18.84% in the western regions and 35.77% in 1^st^ tier cities.

Compared with European and American countries, the use rate of SABA in children with asthma in China is obviously lower, with remarkable differences among different age groups and regions. Therefore, awareness of the importance of SABA still needs to be raised and education and popularization of print and electronic AAP must be increased in order to control asthma attacks in a timely and effective manner.

#### Systemic administration of corticosteroids (SCS)

SCS is a first-line drug for the treatment of severe asthma and acute attacks of severe asthma in children. Patients who have poor efficacy in inhaling high-dose hormones or have a history of oral hormones or a history of critical asthma attacks are often given oral corticosteroids (OCS) or intravenous infusion of corticosteroids, which can prevent the disease from worsening, reduce hospitalization, and even decrease mortality [22]. However, prolonged use of OCS (> 2 weeks) has many side effects, and studies have reported that, even a short-term use of OCS may induce side effects, such as anxiety, mania, irritability and/or aggressive behavior [23, 24]. Studies in adult patients have shown that repeated short-term use of OCS will reduce bone density and increase the incidence of fractures [25, 26]. In addition, the genetic polymorphism of short-term use of OCS adversely affecting bone density in children has been demonstrated [27]. Therefore, in recent years, there is an increasing concern over the excessive use of OCS prescription among children with asthma [28].

According to a study by Farber et al. [29], the prescription rate of OCS (≥ 1 time) for children with asthma aged 1–18 years in Texas, USA, decreased from 34.6% to 17.7% between 2012 and 2015. Among them, a considerable number of OCS were viewed as over-prescribed, and the OCS prescription rate for children aged from 1 to 4 years old (19.0%) was slightly higher than that for children over 4 years old (18.4%). In the Netherlands, the OCS prescription rate in early childhood (25.70%) was relatively higher [30]. In the present study, the overall use rate of OCS was 26.13%, that of infants and young children was 36.00%, and that of central region and tier 3 cities were 58.30% and 56.41%, respectively. Compared with European and American countries, the early OCS prescription rate among children with asthma in China was obviously higher, especially in the central region and tier 3 and below cities. Given the potential side effects of short-term application of OCS, clinicians should be more cautious in prescribing OCS and adhering to the guidelines to treat childhood asthma.

#### Short-acting anticholinergic drugs (SAAC)

SAAC is a component of combination therapy for acute asthma attacks in children and can increase bronchodilation effects, especially in children with moderate to severe asthma who are not responsive to SABA. Pollock et al*.* have shown that, for children of severe asthma, the combination therapy of SABA and SAAC decreased the hospitalization rate by 27% and 74%, respectively, compared with SABA and SAAC alone [31]. A study by Rowe et al. reported that the SAAC use rate in Canada was 77% in the first hour of emergency treatment [32]. A cross-sectional survey from the Auckland community in New Zealand has shown that SAAC usage is very low at only 2.23% [33]. In the present study, the overall SAAC use rate was 55.23%, and the figures were 74.49% for infants and toddlers, 64.63% for the eastern region and 64.59% for 2^nd^ tier cities. As is shown in Table 5, the use of SAAC in general hospitals is higher than that in specialized hospitals. The use of SAAC in tertiary hospitals is higher than that in other hospital grades. Those factors might increase the whole percentage of SAAC use.

To sum up, the use of SAAC varies widely from country to country and from region to region, which may be related to differences in the understanding of the effectiveness of SAAC combination therapy, characteristics of sample hospitals, variations in healthcare and social-economic levels. Gradually improving the understanding of the effectiveness of SAAC combination therapy and narrowing the gaps in medical care among different groups and regions of patients would increase the role of SAAC in the treatment of acute asthma attacks in children.

#### Theophylline

The combination of theophylline and glucocorticoids is often employed for the long-term control of moderate to severe asthma, which can facilitate asthma control and reduce hormone doses. However, theophylline is not as effective as low-dose ICS [34]. Its therapeutic window is relatively narrow, and the toxicity is relatively severe. It is generally not the first choice, and it is not recommended to be used intravenously [13, 18].

Based on the two epidemiological investigations of childhood asthma in China, theophylline use rate has decreased significantly (from 64.0% to 22.4%), which indicates that Chinese clinicians have gradually realized the shortcomings of the weak therapeutic effects and side effects of this agent. In the present study, the overall theophylline use rate was not high (14.38%), but the rates remained high in the central region (63.23%) and in tier 4 and below cities (55.10%). In addition, the proportion of intravenous theophylline used in 4^th^ tier and below cities was up to 50.64%. These data indicate that the side effects of theophylline have not attracted the attention of pediatricians in those regions. It is necessary to further strengthen the training of pediatricians in those corresponding regions and to reduce the use of theophylline, in order to improve the safety of medication for children with asthma.

### Advantages and limitations

To the best of our knowledge, this is the first study based on healthcare insurance data in China for the analysis of asthma medication use among children. The major strengths included its wide spectrum of data coverage and a certain representativeness of the samples. However, the lack of details of clinical data related to the disease, such as manifestation, diagnosis and treatment progress, makes it impossible to perform a more detailed analysis of drug use based on the clinical diagnosis, stage of asthma and treatment of outcomes. In addition, according to China's data protection regulations, we could only use random samples from fixed ratios drawn by provinces, autonomous regions, and municipalities for analyses. Furthermore, since this is a cross-sectional study, we can’t clearly analyze the underlying reasons that have caused the differences of asthma medication use among children in different age groups, regions and levels of urban cities, such as the population density of the city, level of poverty or education, medical resources and physician training.

## Conclusion

The promotion of GINA programs in China has been ongoing for decades and has largely facilitated the standardized treatment of bronchial asthma in children in China. However, there are still remarkable differences in the implementation of GINA programs in children with asthma in different age groups, regions and city levels. Major efforts are still needed to strengthen the education and popularization of the GINA programs and China's guidelines for asthma diagnosis and treatment, especially in the Central and West China, so as to promote the standardized treatment of pediatric asthma and maximize the complete control of childhood asthma in China.

## Supplementary Information

**Additional file 1. Supplementary Table 1.** The drugs’ list to treat asthma.

**Additional file 2. Supplementary Table 2.** Therapeutic combination models.

## Data Availability

The datasets used and/or analyzed during the current study are available from the corresponding author on reasonable request.

## Abbreviations

CHIRA: China Healthcare Insurance Research Association
GINA: Global Initiative for Asthma
DALYs: Daily Adjusted Life Years
ICS: Inhaled Corticosteroids
LABA: Long-acting β2 Receptor Agonist
LTRA: Leukotriene Receptor Antagonist
SABA: Short-acting β2 Receptor Agonist
SCS: Systemic Administration of Corticosteroids
SAAC: Short-acting Anticholinergic Drugs
