# Supplementary material for: Characteristics of medicine use for children with asthma in China: a nationwide population-based study
Source: BMC Pediatr. 2022 Dec 28;22:740. doi: 10.1186/s12887-022-03720-5 (PMC9795755; doi:10.1186/s12887-022-03720-5)
Supplement: Supplementary file 1 — Additional file 1. Supplementary Table 1. The drugs’ list to treat asthma. [file 12887_2022_3720_MOESM1_ESM.docx]

**Supplementary Table 1. The drugs’ list to treat asthma**

| Type Abbreviation | Type Name | Western Medicine Name | [Method](file:///C:\\Users\\Administrator\\AppData\\Local\\Youdao\\Dict\\Application\\7.5.2.0\\resultui\\dict\\?keyword=method)[of](file:///C:\\Users\\Administrator\\AppData\\Local\\Youdao\\Dict\\Application\\7.5.2.0\\resultui\\dict\\?keyword=of)[Administration](file:///C:\\Users\\Administrator\\AppData\\Local\\Youdao\\Dict\\Application\\7.5.2.0\\resultui\\dict\\?keyword=administration) |
| --- | --- | --- | --- |
| GBA | Systemicβ2 receptor agonist | Ammonium bromide | Oral |
| GBA | Systemicβ2 receptor agonist | Ammonium bromide | [Inhalation](C:/Users/Administrator/AppData/Local/Youdao/Dict/Application/6.3.69.8341/resultui/frame/javascript:void(0);) |
| GBA | Systemicβ2 receptor agonist | [Bambuterol](C:/Users/Administrator/AppData/Local/Youdao/Dict/Application/6.3.69.8341/resultui/frame/javascript:void(0);) | Oral |
| GBA | Systemicβ2 receptor agonist | [Bambuterol](C:/Users/Administrator/AppData/Local/Youdao/Dict/Application/6.3.69.8341/resultui/frame/javascript:void(0);) | [Inhalation](C:/Users/Administrator/AppData/Local/Youdao/Dict/Application/6.3.69.8341/resultui/frame/javascript:void(0);) |
| GBA | Systemicβ2 receptor agonist | [Procaterol](C:/Users/Administrator/AppData/Local/Youdao/Dict/Application/6.3.69.8341/resultui/frame/javascript:void(0);) | Oral |
| GBA | Systemicβ2 receptor agonist | [Procaterol](C:/Users/Administrator/AppData/Local/Youdao/Dict/Application/6.3.69.8341/resultui/frame/javascript:void(0);) | [Inhalation](C:/Users/Administrator/AppData/Local/Youdao/Dict/Application/6.3.69.8341/resultui/frame/javascript:void(0);) |
| GBA | Systemicβ2 receptor agonist | [Formoterol](C:/Users/Administrator/AppData/Local/Youdao/Dict/Application/6.3.69.8341/resultui/frame/javascript:void(0);) | Oral |
| GBA | Systemicβ2 receptor agonist | [Clenbuterol](C:/Users/Administrator/AppData/Local/Youdao/Dict/Application/6.3.69.8341/resultui/frame/javascript:void(0);) | Oral |
| GBA | Systemic β2 receptor agonist | [Clenbuterol](C:/Users/Administrator/AppData/Local/Youdao/Dict/Application/6.3.69.8341/resultui/frame/javascript:void(0);) | [Inhalation](C:/Users/Administrator/AppData/Local/Youdao/Dict/Application/6.3.69.8341/resultui/frame/javascript:void(0);) |
| GBA | Systemic β2 receptor agonist | Compound Clenbuterol Hydrochloride | Oral |
| GBA | Systemicβ2 receptor agonist | Compound Clenbuterol Hydrochloride | Other |
| GBA | Systemicβ2 receptor agonist | [Clorprenaline](C:/Users/Administrator/AppData/Local/Youdao/Dict/Application/6.3.69.8341/resultui/frame/javascript:void(0);) | Oral |
| GBA | Systemicβ2 receptor agonist | [Clorprenaline](C:/Users/Administrator/AppData/Local/Youdao/Dict/Application/6.3.69.8341/resultui/frame/javascript:void(0);) | [Inhalation](C:/Users/Administrator/AppData/Local/Youdao/Dict/Application/6.3.69.8341/resultui/frame/javascript:void(0);) |
| GBA | Systemicβ2 receptor agonist | Compound chloropropyrine sodium houttuynia sodium | Oral |
| GBA | Systemicβ2 receptor agonist | Compound chlorpropanol [bromhexine](C:/Users/Administrator/AppData/Local/Youdao/Dict/Application/6.3.69.8341/resultui/frame/javascript:void(0);) | Oral |
| GBA | Systemicβ2 receptor agonist | [Salbutamol](C:/Users/Administrator/AppData/Local/Youdao/Dict/Application/6.3.69.8341/resultui/frame/javascript:void(0);) | Oral |
| GBA | Systemicβ2 receptor agonist | [Salbutamol](C:/Users/Administrator/AppData/Local/Youdao/Dict/Application/6.3.69.8341/resultui/frame/javascript:void(0);) | [Inhalation](C:/Users/Administrator/AppData/Local/Youdao/Dict/Application/6.3.69.8341/resultui/frame/javascript:void(0);) |
| GBA | Systemicβ2 receptor agonist | [Terbutaline](C:/Users/Administrator/AppData/Local/Youdao/Dict/Application/6.3.69.8341/resultui/frame/javascript:void(0);) | [Inhalation](C:/Users/Administrator/AppData/Local/Youdao/Dict/Application/6.3.69.8341/resultui/frame/javascript:void(0);) |
| GBA | Systemicβ2 receptor agonist | [Terbutaline](C:/Users/Administrator/AppData/Local/Youdao/Dict/Application/6.3.69.8341/resultui/frame/javascript:void(0);) | Oral |
| GBA | Systemicβ2 receptor agonist | Tebutalin sodium chloride | [Inhalation](C:/Users/Administrator/AppData/Local/Youdao/Dict/Application/6.3.69.8341/resultui/frame/javascript:void(0);) |
| GBA | Systemicβ2 receptor agonist | Tulobuterol | [Patch](C:/Users/Administrator/AppData/Local/Youdao/Dict/Application/6.3.69.8341/resultui/frame/javascript:void(0);) |
| GBA | Systemicβ2 receptor agonist | Children's compound tolotrow | Oral |
| GBA | Systemicβ2 receptor agonist | Children's compound tolotrow | [Inhalation](C:/Users/Administrator/AppData/Local/Youdao/Dict/Application/6.3.69.8341/resultui/frame/javascript:void(0);) |
| GBA | Systemicβ2 receptor agonist | Isoprenaline | Oral |
| GBA | Systemicβ2 receptor agonist | [Hexoprenaline](C:/Users/Administrator/AppData/Local/Youdao/Dict/Application/6.3.69.8341/resultui/frame/javascript:void(0);) | Oral |
| GBA | Systemicβ2 receptor agonist | Cycloclenbuterol | Oral |
| GBA | Systemicβ2 receptor agonist | Children compound ephedrine platycodon | Oral |
| GC | Systemic glucocorticoid | [Betamethasone](C:/Users/Administrator/AppData/Local/Youdao/Dict/Application/6.3.69.8341/resultui/frame/javascript:void(0);) | Oral |
| GC | Systemic glucocorticoid | [Betamethasone](C:/Users/Administrator/AppData/Local/Youdao/Dict/Application/6.3.69.8341/resultui/frame/javascript:void(0);) | [Inhalation](C:/Users/Administrator/AppData/Local/Youdao/Dict/Application/6.3.69.8341/resultui/frame/javascript:void(0);) |
| GC | Systemic glucocorticoid | [Dexamethasone](C:/Users/Administrator/AppData/Local/Youdao/Dict/Application/6.3.69.8341/resultui/frame/javascript:void(0);) | Oral |
| GC | Systemic glucocorticoid | [Dexamethasone](C:/Users/Administrator/AppData/Local/Youdao/Dict/Application/6.3.69.8341/resultui/frame/javascript:void(0);) | [Inhalation](C:/Users/Administrator/AppData/Local/Youdao/Dict/Application/6.3.69.8341/resultui/frame/javascript:void(0);) |
| GC | Systemic glucocorticoid | [Dexamethasone](C:/Users/Administrator/AppData/Local/Youdao/Dict/Application/6.3.69.8341/resultui/frame/javascript:void(0);) [sodium](C:/Users/Administrator/AppData/Local/Youdao/Dict/Application/6.3.69.8341/resultui/frame/javascript:void(0);) [phosphate](C:/Users/Administrator/AppData/Local/Youdao/Dict/Application/6.3.69.8341/resultui/frame/javascript:void(0);) | [Inhalation](C:/Users/Administrator/AppData/Local/Youdao/Dict/Application/6.3.69.8341/resultui/frame/javascript:void(0);) |
| GC | Systemic glucocorticoid | Dexamethasone palmitate | [Inhalation](C:/Users/Administrator/AppData/Local/Youdao/Dict/Application/6.3.69.8341/resultui/frame/javascript:void(0);) |
| GC | Systemic glucocorticoid | Compound betamethasone | [Inhalation](C:/Users/Administrator/AppData/Local/Youdao/Dict/Application/6.3.69.8341/resultui/frame/javascript:void(0);) |
| GC | Systemic glucocorticoid | Compound betamethasone | Oral |
| GC | Systemic glucocorticoid | [Methylprednisolone](C:/Users/Administrator/AppData/Local/Youdao/Dict/Application/6.3.69.8341/resultui/frame/javascript:void(0);) | Oral |
| GC | Systemic glucocorticoid | [Methylprednisolone](C:/Users/Administrator/AppData/Local/Youdao/Dict/Application/6.3.69.8341/resultui/frame/javascript:void(0);) | [Inhalation](C:/Users/Administrator/AppData/Local/Youdao/Dict/Application/6.3.69.8341/resultui/frame/javascript:void(0);) |
| GC | Systemic glucocorticoid | [Cortisone](C:/Users/Administrator/AppData/Local/Youdao/Dict/Application/6.3.69.8341/resultui/frame/javascript:void(0);) | Oral |
| GC | Systemic glucocorticoid | [Cortisone](C:/Users/Administrator/AppData/Local/Youdao/Dict/Application/6.3.69.8341/resultui/frame/javascript:void(0);) | [Inhalation](C:/Users/Administrator/AppData/Local/Youdao/Dict/Application/6.3.69.8341/resultui/frame/javascript:void(0);) |
| GC | Systemic glucocorticoid | Prednisone | Oral |
| GC | Systemic glucocorticoid | Prednisone | [Inhalation](C:/Users/Administrator/AppData/Local/Youdao/Dict/Application/6.3.69.8341/resultui/frame/javascript:void(0);) |
| GC | Systemic glucocorticoid | [Prednisolone](C:/Users/Administrator/AppData/Local/Youdao/Dict/Application/6.3.69.8341/resultui/frame/javascript:void(0);) | Oral |
| GC | Systemic glucocorticoid | [Prednisolone](C:/Users/Administrator/AppData/Local/Youdao/Dict/Application/6.3.69.8341/resultui/frame/javascript:void(0);) | [Inhalation](C:/Users/Administrator/AppData/Local/Youdao/Dict/Application/6.3.69.8341/resultui/frame/javascript:void(0);) |
| GC | Systemic glucocorticoid | Hydrocortisone | Oral |
| GC | Systemic glucocorticoid | Hydrocortisone | [Inhalation](C:/Users/Administrator/AppData/Local/Youdao/Dict/Application/6.3.69.8341/resultui/frame/javascript:void(0);) |
| GC | Systemic glucocorticoid | Hydrocortisone | Oral |
| GC | Systemic glucocorticoid | Hydrocortisone | [Inhalation](C:/Users/Administrator/AppData/Local/Youdao/Dict/Application/6.3.69.8341/resultui/frame/javascript:void(0);) |
| GC | Systemic glucocorticoid | Triamcinolone acetonide | [Inhalation](C:/Users/Administrator/AppData/Local/Youdao/Dict/Application/6.3.69.8341/resultui/frame/javascript:void(0);) |
| GC | Systemic glucocorticoid | Triamcinolone acetonide | Oral |
| GC | Systemic glucocorticoid | [Yriamcinolone](C:/Users/Administrator/AppData/Local/Youdao/Dict/Application/6.3.69.8341/resultui/frame/javascript:void(0);) | Oral |
| ICS | Inhaled corticosteroids | [Beclomethasone](C:/Users/Administrator/AppData/Local/Youdao/Dict/Application/6.3.69.8341/resultui/frame/javascript:void(0);) | [Inhalation](C:/Users/Administrator/AppData/Local/Youdao/Dict/Application/6.3.69.8341/resultui/frame/javascript:void(0);) |
| ICS | Inhaled corticosteroids | [Budesonide](C:/Users/Administrator/AppData/Local/Youdao/Dict/Application/6.3.69.8341/resultui/frame/javascript:void(0);) | [Inhalation](C:/Users/Administrator/AppData/Local/Youdao/Dict/Application/6.3.69.8341/resultui/frame/javascript:void(0);) |
| ICS | Inhaled corticosteroids | [Budesonide](C:/Users/Administrator/AppData/Local/Youdao/Dict/Application/6.3.69.8341/resultui/frame/javascript:void(0);) | [nebulization](C:/Users/Administrator/AppData/Local/Youdao/Dict/Application/6.3.69.8341/resultui/frame/javascript:void(0);) |
| ICS | Inhaled corticosteroids | [Fluticasone](C:/Users/Administrator/AppData/Local/Youdao/Dict/Application/6.3.69.8341/resultui/frame/javascript:void(0);) | [Inhalation](C:/Users/Administrator/AppData/Local/Youdao/Dict/Application/6.3.69.8341/resultui/frame/javascript:void(0);) |
| ICS | Inhaled corticosteroids | [Ciclesonide](C:/Users/Administrator/AppData/Local/Youdao/Dict/Application/6.3.69.8341/resultui/frame/javascript:void(0);) | [Inhalation](C:/Users/Administrator/AppData/Local/Youdao/Dict/Application/6.3.69.8341/resultui/frame/javascript:void(0);) |
| ICS | Inhaled corticosteroids | [Mometasone](C:/Users/Administrator/AppData/Local/Youdao/Dict/Application/6.3.69.8341/resultui/frame/javascript:void(0);) [furoate](C:/Users/Administrator/AppData/Local/Youdao/Dict/Application/6.3.69.8341/resultui/frame/javascript:void(0);) | [Inhalation](C:/Users/Administrator/AppData/Local/Youdao/Dict/Application/6.3.69.8341/resultui/frame/javascript:void(0);) |
| ICS | Inhaled corticosteroids | Triamcinolone acetonide | [Inhalation](C:/Users/Administrator/AppData/Local/Youdao/Dict/Application/6.3.69.8341/resultui/frame/javascript:void(0);) |
| ICS | Inhaled corticosteroids | [Mometasone](C:/Users/Administrator/AppData/Local/Youdao/Dict/Application/6.3.69.8341/resultui/frame/javascript:void(0);) | [Inhalation](C:/Users/Administrator/AppData/Local/Youdao/Dict/Application/6.3.69.8341/resultui/frame/javascript:void(0);) |
| ICS/LABA | Inhaled corticosteroids /  Long-actingβ2 agonists | Betamethasone fumotero | [Inhalation](C:/Users/Administrator/AppData/Local/Youdao/Dict/Application/6.3.69.8341/resultui/frame/javascript:void(0);) |
| ICS/LABA | Inhaled corticosteroids /  Long-actingβ2 agonists | Budesonide formotero | [Inhalation](C:/Users/Administrator/AppData/Local/Youdao/Dict/Application/6.3.69.8341/resultui/frame/javascript:void(0);) |
| ICS/LABA | Inhaled corticosteroids /  Long-actingβ2 agonists | Fluticasone salamisol | [Inhalation](C:/Users/Administrator/AppData/Local/Youdao/Dict/Application/6.3.69.8341/resultui/frame/javascript:void(0);) |
| ICS/LABA | Inhaled corticosteroids /  Long-actingβ2 agonists | Salmeterotikacin | [Inhalation](C:/Users/Administrator/AppData/Local/Youdao/Dict/Application/6.3.69.8341/resultui/frame/javascript:void(0);) |
| LABA | Long-actingβ2 agonists | [Formoterol](C:/Users/Administrator/AppData/Local/Youdao/Dict/Application/6.3.69.8341/resultui/frame/javascript:void(0);) | [Inhalation](C:/Users/Administrator/AppData/Local/Youdao/Dict/Application/6.3.69.8341/resultui/frame/javascript:void(0);) |
| LABA | Long-actingβ2 agonists | [Clenbuterol](C:/Users/Administrator/AppData/Local/Youdao/Dict/Application/6.3.69.8341/resultui/frame/javascript:void(0);) | [Inhalation](C:/Users/Administrator/AppData/Local/Youdao/Dict/Application/6.3.69.8341/resultui/frame/javascript:void(0);) |
| LABA | Long-actingβ2 agonists | [Salmeterol](C:/Users/Administrator/AppData/Local/Youdao/Dict/Application/6.3.69.8341/resultui/frame/javascript:void(0);) | [Inhalation](C:/Users/Administrator/AppData/Local/Youdao/Dict/Application/6.3.69.8341/resultui/frame/javascript:void(0);) |
| LABA | Long-actingβ2 agonists | Indacaterol | [Inhalation](C:/Users/Administrator/AppData/Local/Youdao/Dict/Application/6.3.69.8341/resultui/frame/javascript:void(0);) |
| LABA | Long-actingβ2 agonists | Carbochromen | [Inhalation](C:/Users/Administrator/AppData/Local/Youdao/Dict/Application/6.3.69.8341/resultui/frame/javascript:void(0);) |
| LAMA | [Long-acting anticholinergic](javascript:;) | [Tiotropium](C:/Users/Administrator/AppData/Local/Youdao/Dict/Application/6.3.69.8341/resultui/frame/javascript:void(0);) [Bromide](C:/Users/Administrator/AppData/Local/Youdao/Dict/Application/6.3.69.8341/resultui/frame/javascript:void(0);) | [Inhalation](C:/Users/Administrator/AppData/Local/Youdao/Dict/Application/6.3.69.8341/resultui/frame/javascript:void(0);) |
| LTRA | Leukotriene receptor antagonist | [Montelukast](C:/Users/Administrator/AppData/Local/Youdao/Dict/Application/6.3.69.8341/resultui/frame/javascript:void(0);) | Oral |
| LTRA | Leukotriene receptor antagonist | [Ibudilast](C:/Users/Administrator/AppData/Local/Youdao/Dict/Application/6.3.69.8341/resultui/frame/javascript:void(0);) | Oral |
| LTRA | Leukotriene receptor antagonist | [Zafirlukast](C:/Users/Administrator/AppData/Local/Youdao/Dict/Application/6.3.69.8341/resultui/frame/javascript:void(0);) | Oral |
| SABA | Short-actingβ2 agonists | [Bambuterol](C:/Users/Administrator/AppData/Local/Youdao/Dict/Application/6.3.69.8341/resultui/frame/javascript:void(0);) | [Inhalation](C:/Users/Administrator/AppData/Local/Youdao/Dict/Application/6.3.69.8341/resultui/frame/javascript:void(0);) |
| SABA | Short-actingβ2 agonists | [Salbutamol](C:/Users/Administrator/AppData/Local/Youdao/Dict/Application/6.3.69.8341/resultui/frame/javascript:void(0);) | [Inhalation](C:/Users/Administrator/AppData/Local/Youdao/Dict/Application/6.3.69.8341/resultui/frame/javascript:void(0);) |
| SABA | Short-actingβ2 agonists | [Salbutamol](C:/Users/Administrator/AppData/Local/Youdao/Dict/Application/6.3.69.8341/resultui/frame/javascript:void(0);) | [Nebulization](C:/Users/Administrator/AppData/Local/Youdao/Dict/Application/6.3.69.8341/resultui/frame/javascript:void(0);) |
| SABA | Short-actingβ2 agonists | [Terbutaline](C:/Users/Administrator/AppData/Local/Youdao/Dict/Application/6.3.69.8341/resultui/frame/javascript:void(0);) | [Inhalation](C:/Users/Administrator/AppData/Local/Youdao/Dict/Application/6.3.69.8341/resultui/frame/javascript:void(0);) |
| SABA | Short-actingβ2 agonists | [Terbutaline](C:/Users/Administrator/AppData/Local/Youdao/Dict/Application/6.3.69.8341/resultui/frame/javascript:void(0);) | [Nebulization](C:/Users/Administrator/AppData/Local/Youdao/Dict/Application/6.3.69.8341/resultui/frame/javascript:void(0);) |
| SABA | Short-actingβ2 agonists | [Isoprenaline](C:/Users/Administrator/AppData/Local/Youdao/Dict/Application/6.3.69.8341/resultui/frame/javascript:void(0);) | [Inhalation](C:/Users/Administrator/AppData/Local/Youdao/Dict/Application/6.3.69.8341/resultui/frame/javascript:void(0);) |
| SABA | Short-actingβ2 agonists | Isoproterenol hydrochloride | [Inhalation](C:/Users/Administrator/AppData/Local/Youdao/Dict/Application/6.3.69.8341/resultui/frame/javascript:void(0);) |
| SABA/SAMA | Short-actingβ2 agonist+ Short-acting Anticholinergic agonist | [Ipratropium](file:///C:\\Users\\Administrator\\AppData\\Local\\Youdao\\Dict\\Application\\7.5.2.0\\resultui\\dict\\?keyword=ipratropium)[bromide](file:///C:\\Users\\Administrator\\AppData\\Local\\Youdao\\Dict\\Application\\7.5.2.0\\resultui\\dict\\?keyword=bromide) | [Inhalation](C:/Users/Administrator/AppData/Local/Youdao/Dict/Application/6.3.69.8341/resultui/frame/javascript:void(0);) |
| SABA/SAMA | Short-acting β2 agonist+ Short-acting Anticholinergic agonist | [Ipratropium](file:///C:\\Users\\Administrator\\AppData\\Local\\Youdao\\Dict\\Application\\7.5.2.0\\resultui\\dict\\?keyword=ipratropium)[bromide](file:///C:\\Users\\Administrator\\AppData\\Local\\Youdao\\Dict\\Application\\7.5.2.0\\resultui\\dict\\?keyword=bromide) | [Nebulization](C:/Users/Administrator/AppData/Local/Youdao/Dict/Application/6.3.69.8341/resultui/frame/javascript:void(0);) |
| SAMA | Short-acting Anticholinergic agonist | [Ipratropium](file:///C:\\Users\\Administrator\\AppData\\Local\\Youdao\\Dict\\Application\\7.5.2.0\\resultui\\dict\\?keyword=ipratropium)[bromide](file:///C:\\Users\\Administrator\\AppData\\Local\\Youdao\\Dict\\Application\\7.5.2.0\\resultui\\dict\\?keyword=bromide) | [Nebulization](C:/Users/Administrator/AppData/Local/Youdao/Dict/Application/6.3.69.8341/resultui/frame/javascript:void(0);) |
| SAMA | Short-acting Anticholinergic agonist | [Ipratropium](file:///C:\\Users\\Administrator\\AppData\\Local\\Youdao\\Dict\\Application\\7.5.2.0\\resultui\\dict\\?keyword=ipratropium)[bromide](file:///C:\\Users\\Administrator\\AppData\\Local\\Youdao\\Dict\\Application\\7.5.2.0\\resultui\\dict\\?keyword=bromide) | [Inhalation](C:/Users/Administrator/AppData/Local/Youdao/Dict/Application/6.3.69.8341/resultui/frame/javascript:void(0);) |
| SAMA | Short-acting Anticholinergic agonist | [Pemirolast](C:/Users/Administrator/AppData/Local/Youdao/Dict/Application/6.3.69.8341/resultui/frame/javascript:void(0);) | Oral |
| SAMA | Short-acting Anticholinergic agonist | Compound Tranilast | Oral |
| SAMA | Short-acting Anticholinergic agonist | [Suplatast](C:/Users/Administrator/AppData/Local/Youdao/Dict/Application/6.3.69.8341/resultui/frame/javascript:void(0);) [Tosilate](C:/Users/Administrator/AppData/Local/Youdao/Dict/Application/6.3.69.8341/resultui/frame/javascript:void(0);) | Oral |
| SAMA | Short-acting Anticholinergic agonist | [Tranilast](C:/Users/Administrator/AppData/Local/Youdao/Dict/Application/6.3.69.8341/resultui/frame/javascript:void(0);) | Oral |
| SAMA | Short-acting Anticholinergic agonist | Seratrodast | Oral |
| SAMA | Short-acting Anticholinergic agonist | [Ketotifen](C:/Users/Administrator/AppData/Local/Youdao/Dict/Application/6.3.69.8341/resultui/frame/javascript:void(0);) | Oral |
| SAMA | Short-acting Anticholinergic agonist | [Ketotifen](C:/Users/Administrator/AppData/Local/Youdao/Dict/Application/6.3.69.8341/resultui/frame/javascript:void(0);) | [Inhalation](C:/Users/Administrator/AppData/Local/Youdao/Dict/Application/6.3.69.8341/resultui/frame/javascript:void(0);) |
| Theophilline | Xanthines | [Aminophylline](C:/Users/Administrator/AppData/Local/Youdao/Dict/Application/6.3.69.8341/resultui/frame/javascript:void(0);) | Oral |
| Theophilline | Xanthines | [Aminophylline](C:/Users/Administrator/AppData/Local/Youdao/Dict/Application/6.3.69.8341/resultui/frame/javascript:void(0);) | [Injection](C:/Users/Administrator/AppData/Local/Youdao/Dict/Application/6.3.69.8341/resultui/frame/javascript:void(0);) |
| Theophilline | Xanthines | Compound toluene ephedrine | Oral |
| Theophilline | Xanthines | Compound toluene ephedrine | [Injection](C:/Users/Administrator/AppData/Local/Youdao/Dict/Application/6.3.69.8341/resultui/frame/javascript:void(0);) |
| Theophilline | Xanthines | Compound dioxalazine theophylline | Oral |
| Theophilline | Xanthines | Compound dioxalazine Theophylline | [Injection](C:/Users/Administrator/AppData/Local/Youdao/Dict/Application/6.3.69.8341/resultui/frame/javascript:void(0);) |
| Theophilline | Xanthines | Theophylline | Oral |
| Theophilline | Xanthines | Theophylline | [Injection](C:/Users/Administrator/AppData/Local/Youdao/Dict/Application/6.3.69.8341/resultui/frame/javascript:void(0);) |
| Theophilline | Xanthines | TheophyllineⅡ | Oral |
| Theophilline | Xanthines | TheophyllineⅡ | [Injection](C:/Users/Administrator/AppData/Local/Youdao/Dict/Application/6.3.69.8341/resultui/frame/javascript:void(0);) |
| Theophilline | Xanthines | Compound theophylline ketofen | Oral |
| Theophilline | Xanthines | Compound theophylline ketofen | [Injection](C:/Users/Administrator/AppData/Local/Youdao/Dict/Application/6.3.69.8341/resultui/frame/javascript:void(0);) |
| Theophilline | Xanthines | [Choline](C:/Users/Administrator/AppData/Local/Youdao/Dict/Application/6.3.69.8341/resultui/frame/javascript:void(0);) [theophyllinate](C:/Users/Administrator/AppData/Local/Youdao/Dict/Application/6.3.69.8341/resultui/frame/javascript:void(0);) | Oral |
| Theophilline | Xanthines | [Choline](C:/Users/Administrator/AppData/Local/Youdao/Dict/Application/6.3.69.8341/resultui/frame/javascript:void(0);) [theophyllinate](C:/Users/Administrator/AppData/Local/Youdao/Dict/Application/6.3.69.8341/resultui/frame/javascript:void(0);) | [Injection](C:/Users/Administrator/AppData/Local/Youdao/Dict/Application/6.3.69.8341/resultui/frame/javascript:void(0);) |
| Theophilline | Xanthines | [Doxofylline](C:/Users/Administrator/AppData/Local/Youdao/Dict/Application/6.3.69.8341/resultui/frame/javascript:void(0);) | [Injection](C:/Users/Administrator/AppData/Local/Youdao/Dict/Application/6.3.69.8341/resultui/frame/javascript:void(0);) |
| Theophilline | Xanthines | [Doxofylline](C:/Users/Administrator/AppData/Local/Youdao/Dict/Application/6.3.69.8341/resultui/frame/javascript:void(0);) | Oral |
| Theophilline | Xanthines | Doxylline sodium chloride | [Injection](C:/Users/Administrator/AppData/Local/Youdao/Dict/Application/6.3.69.8341/resultui/frame/javascript:void(0);) |
| Theophilline | Xanthines | Polytheophylline glucose | [Injection](C:/Users/Administrator/AppData/Local/Youdao/Dict/Application/6.3.69.8341/resultui/frame/javascript:void(0);) |
| Theophilline | Xanthines | [Diprophylline](C:/Users/Administrator/AppData/Local/Youdao/Dict/Application/6.3.69.8341/resultui/frame/javascript:void(0);) | Oral |
| Theophilline | Xanthines | [Diprophylline](C:/Users/Administrator/AppData/Local/Youdao/Dict/Application/6.3.69.8341/resultui/frame/javascript:void(0);) | [Injection](C:/Users/Administrator/AppData/Local/Youdao/Dict/Application/6.3.69.8341/resultui/frame/javascript:void(0);) |
| Theophilline | Xanthines | Dihydroxypropylline sodium chloride | [Injection](C:/Users/Administrator/AppData/Local/Youdao/Dict/Application/6.3.69.8341/resultui/frame/javascript:void(0);) |
| Theophilline | Xanthines | Aminophylline combination | Oral |
| Theophilline | Xanthines | Aminophylline combination | [Injection](C:/Users/Administrator/AppData/Local/Youdao/Dict/Application/6.3.69.8341/resultui/frame/javascript:void(0);) |
| Theophilline | Xanthines | Compound aminophylline storm horse | Oral |
| Theophilline | Xanthines | Compound aminophylline storm horse | [Injection](C:/Users/Administrator/AppData/Local/Youdao/Dict/Application/6.3.69.8341/resultui/frame/javascript:void(0);) |
| Theophilline | Xanthines | Compound aminopyrine theophylline | Oral |
| Theophilline | Xanthines | Compound aminopyrine theophylline | [Injection](C:/Users/Administrator/AppData/Local/Youdao/Dict/Application/6.3.69.8341/resultui/frame/javascript:void(0);) |
| Theophilline | Xanthines | Compound theophylline methamphetamine | Oral |
| Theophilline | Xanthines | Compound theophylline methamphetamine | [Injection](C:/Users/Administrator/AppData/Local/Youdao/Dict/Application/6.3.69.8341/resultui/frame/javascript:void(0);) |
| Theophilline | Xanthines | Compound theophylline methamphetamine | Oral |
| Theophilline | Xanthines | Compound theophylline methamphetamine | [Injection](C:/Users/Administrator/AppData/Local/Youdao/Dict/Application/6.3.69.8341/resultui/frame/javascript:void(0);) |
| Theophilline | Xanthines | Compound bile ammonia | Oral |
| Theophilline | Xanthines | Compound bile ammonia | Oral |
| Theophilline | Xanthines | Compound glycine theophylline | Oral |
| Theophilline | Xanthines | Compound glycine theophylline | [Injection](C:/Users/Administrator/AppData/Local/Youdao/Dict/Application/6.3.69.8341/resultui/frame/javascript:void(0);) |
| Theophilline | Xanthines | Compound hydroxyprophylline Decloxizine | Oral |
| Theophilline | Xanthines | Compound hydroxyprophylline Decloxizine | [Injection](C:/Users/Administrator/AppData/Local/Youdao/Dict/Application/6.3.69.8341/resultui/frame/javascript:void(0);) |
| Theophilline | Xanthines | Glycine theophylline | Oral |
| Theophilline | Xanthines | Glycine theophylline | [Injection](C:/Users/Administrator/AppData/Local/Youdao/Dict/Application/6.3.69.8341/resultui/frame/javascript:void(0);) |
| Theophilline | Xanthines | Aminophylline sodium chloride | [Injection](C:/Users/Administrator/AppData/Local/Youdao/Dict/Application/6.3.69.8341/resultui/frame/javascript:void(0);) |
| Theophilline | Xanthines | Theophylline glucose | [Injection](C:/Users/Administrator/AppData/Local/Youdao/Dict/Application/6.3.69.8341/resultui/frame/javascript:void(0);) |
| Theophilline | Xanthines | Theophylline albuterol | Oral |
| Theophilline | Xanthines | Compound theophyline | Oral |
| Theophilline | Xanthines | Compound theophyline | [Injection](C:/Users/Administrator/AppData/Local/Youdao/Dict/Application/6.3.69.8341/resultui/frame/javascript:void(0);) |
| Theophilline | Xanthines | Theophylline sodium chloride | [Injection](C:/Users/Administrator/AppData/Local/Youdao/Dict/Application/6.3.69.8341/resultui/frame/javascript:void(0);) |
| [Expectorant](C:/Users/Administrator/AppData/Local/Youdao/Dict/Application/6.3.69.8341/resultui/frame/javascript:void(0);) | [Expectorant](C:/Users/Administrator/AppData/Local/Youdao/Dict/Application/6.3.69.8341/resultui/frame/javascript:void(0);) | Ambroxol | Oral |
| [Expectorant](C:/Users/Administrator/AppData/Local/Youdao/Dict/Application/6.3.69.8341/resultui/frame/javascript:void(0);) | [Expectorant](C:/Users/Administrator/AppData/Local/Youdao/Dict/Application/6.3.69.8341/resultui/frame/javascript:void(0);) | Ambroxol | [Injection](C:/Users/Administrator/AppData/Local/Youdao/Dict/Application/6.3.69.8341/resultui/frame/javascript:void(0);) |
| [Expectorant](C:/Users/Administrator/AppData/Local/Youdao/Dict/Application/6.3.69.8341/resultui/frame/javascript:void(0);) | [Expectorant](C:/Users/Administrator/AppData/Local/Youdao/Dict/Application/6.3.69.8341/resultui/frame/javascript:void(0);) | Ambroxol sodium chloride | [Injection](C:/Users/Administrator/AppData/Local/Youdao/Dict/Application/6.3.69.8341/resultui/frame/javascript:void(0);) |
| [Expectorant](C:/Users/Administrator/AppData/Local/Youdao/Dict/Application/6.3.69.8341/resultui/frame/javascript:void(0);) | [Expectorant](C:/Users/Administrator/AppData/Local/Youdao/Dict/Application/6.3.69.8341/resultui/frame/javascript:void(0);) | Ambroxol glucose | [Injection](C:/Users/Administrator/AppData/Local/Youdao/Dict/Application/6.3.69.8341/resultui/frame/javascript:void(0);) |
| [Expectorant](C:/Users/Administrator/AppData/Local/Youdao/Dict/Application/6.3.69.8341/resultui/frame/javascript:void(0);) | [Expectorant](C:/Users/Administrator/AppData/Local/Youdao/Dict/Application/6.3.69.8341/resultui/frame/javascript:void(0);) | [Cysteine](C:/Users/Administrator/AppData/Local/Youdao/Dict/Application/6.3.69.8341/resultui/frame/javascript:void(0);) | Oral |
| [Expectorant](C:/Users/Administrator/AppData/Local/Youdao/Dict/Application/6.3.69.8341/resultui/frame/javascript:void(0);) | [Expectorant](C:/Users/Administrator/AppData/Local/Youdao/Dict/Application/6.3.69.8341/resultui/frame/javascript:void(0);) | [Cysteine](C:/Users/Administrator/AppData/Local/Youdao/Dict/Application/6.3.69.8341/resultui/frame/javascript:void(0);) | [Injection](C:/Users/Administrator/AppData/Local/Youdao/Dict/Application/6.3.69.8341/resultui/frame/javascript:void(0);) |
| [Expectorant](C:/Users/Administrator/AppData/Local/Youdao/Dict/Application/6.3.69.8341/resultui/frame/javascript:void(0);) | [Expectorant](C:/Users/Administrator/AppData/Local/Youdao/Dict/Application/6.3.69.8341/resultui/frame/javascript:void(0);) | [Erdosteine](C:/Users/Administrator/AppData/Local/Youdao/Dict/Application/6.3.69.8341/resultui/frame/javascript:void(0);) | Oral |
| [Expectorant](C:/Users/Administrator/AppData/Local/Youdao/Dict/Application/6.3.69.8341/resultui/frame/javascript:void(0);) | [Expectorant](C:/Users/Administrator/AppData/Local/Youdao/Dict/Application/6.3.69.8341/resultui/frame/javascript:void(0);) | Fudosteine | Oral |
| [Expectorant](C:/Users/Administrator/AppData/Local/Youdao/Dict/Application/6.3.69.8341/resultui/frame/javascript:void(0);) | [Expectorant](C:/Users/Administrator/AppData/Local/Youdao/Dict/Application/6.3.69.8341/resultui/frame/javascript:void(0);) | [Serrapeptase](C:/Users/Administrator/AppData/Local/Youdao/Dict/Application/6.3.69.8341/resultui/frame/javascript:void(0);) | Oral |
| [Expectorant](C:/Users/Administrator/AppData/Local/Youdao/Dict/Application/6.3.69.8341/resultui/frame/javascript:void(0);) | [Expectorant](C:/Users/Administrator/AppData/Local/Youdao/Dict/Application/6.3.69.8341/resultui/frame/javascript:void(0);) | [Carbocysteine](C:/Users/Administrator/AppData/Local/Youdao/Dict/Application/6.3.69.8341/resultui/frame/javascript:void(0);) | Oral |
| [Expectorant](C:/Users/Administrator/AppData/Local/Youdao/Dict/Application/6.3.69.8341/resultui/frame/javascript:void(0);) | [Expectorant](C:/Users/Administrator/AppData/Local/Youdao/Dict/Application/6.3.69.8341/resultui/frame/javascript:void(0);) | [Carbocysteine](C:/Users/Administrator/AppData/Local/Youdao/Dict/Application/6.3.69.8341/resultui/frame/javascript:void(0);) | [Injection](C:/Users/Administrator/AppData/Local/Youdao/Dict/Application/6.3.69.8341/resultui/frame/javascript:void(0);) |
| [Expectorant](C:/Users/Administrator/AppData/Local/Youdao/Dict/Application/6.3.69.8341/resultui/frame/javascript:void(0);) | [Expectorant](C:/Users/Administrator/AppData/Local/Youdao/Dict/Application/6.3.69.8341/resultui/frame/javascript:void(0);) | [Bromhexine](C:/Users/Administrator/AppData/Local/Youdao/Dict/Application/6.3.69.8341/resultui/frame/javascript:void(0);) | Oral |
| [Expectorant](C:/Users/Administrator/AppData/Local/Youdao/Dict/Application/6.3.69.8341/resultui/frame/javascript:void(0);) | [Expectorant](C:/Users/Administrator/AppData/Local/Youdao/Dict/Application/6.3.69.8341/resultui/frame/javascript:void(0);) | [Bromhexine](C:/Users/Administrator/AppData/Local/Youdao/Dict/Application/6.3.69.8341/resultui/frame/javascript:void(0);) | [Injection](C:/Users/Administrator/AppData/Local/Youdao/Dict/Application/6.3.69.8341/resultui/frame/javascript:void(0);) |
| [Expectorant](C:/Users/Administrator/AppData/Local/Youdao/Dict/Application/6.3.69.8341/resultui/frame/javascript:void(0);) | [Expectorant](C:/Users/Administrator/AppData/Local/Youdao/Dict/Application/6.3.69.8341/resultui/frame/javascript:void(0);) | [Bromhexine](C:/Users/Administrator/AppData/Local/Youdao/Dict/Application/6.3.69.8341/resultui/frame/javascript:void(0);) glucose | [Injection](C:/Users/Administrator/AppData/Local/Youdao/Dict/Application/6.3.69.8341/resultui/frame/javascript:void(0);) |
| [Expectorant](C:/Users/Administrator/AppData/Local/Youdao/Dict/Application/6.3.69.8341/resultui/frame/javascript:void(0);) | [Expectorant](C:/Users/Administrator/AppData/Local/Youdao/Dict/Application/6.3.69.8341/resultui/frame/javascript:void(0);) | Acetylcysteine | Oral |
| [Expectorant](C:/Users/Administrator/AppData/Local/Youdao/Dict/Application/6.3.69.8341/resultui/frame/javascript:void(0);) | [Expectorant](C:/Users/Administrator/AppData/Local/Youdao/Dict/Application/6.3.69.8341/resultui/frame/javascript:void(0);) | Acetylcysteine | [Inhalation](C:/Users/Administrator/AppData/Local/Youdao/Dict/Application/6.3.69.8341/resultui/frame/javascript:void(0);) |
| [Expectorant](C:/Users/Administrator/AppData/Local/Youdao/Dict/Application/6.3.69.8341/resultui/frame/javascript:void(0);) | [Expectorant](C:/Users/Administrator/AppData/Local/Youdao/Dict/Application/6.3.69.8341/resultui/frame/javascript:void(0);) | Acetylcysteine | [Injection](C:/Users/Administrator/AppData/Local/Youdao/Dict/Application/6.3.69.8341/resultui/frame/javascript:void(0);) |
| [Expectorant](C:/Users/Administrator/AppData/Local/Youdao/Dict/Application/6.3.69.8341/resultui/frame/javascript:void(0);) | [Expectorant](C:/Users/Administrator/AppData/Local/Youdao/Dict/Application/6.3.69.8341/resultui/frame/javascript:void(0);) | [Mecysteine](C:/Users/Administrator/AppData/Local/Youdao/Dict/Application/6.3.69.8341/resultui/frame/javascript:void(0);) | Oral |
| [Expectorant](C:/Users/Administrator/AppData/Local/Youdao/Dict/Application/6.3.69.8341/resultui/frame/javascript:void(0);) | [Expectorant](C:/Users/Administrator/AppData/Local/Youdao/Dict/Application/6.3.69.8341/resultui/frame/javascript:void(0);) | Neutral protease | Oral |
| [Expectorant](C:/Users/Administrator/AppData/Local/Youdao/Dict/Application/6.3.69.8341/resultui/frame/javascript:void(0);) | [Expectorant](C:/Users/Administrator/AppData/Local/Youdao/Dict/Application/6.3.69.8341/resultui/frame/javascript:void(0);) | Guacetisal | Oral |
| Systemic Antihistamines | Systemic Antihistamines | Acrivastine | Oral |
| Systemic Antihistamines | Systemic Antihistamines | [Antazoline](C:/Users/Administrator/AppData/Local/Youdao/Dict/Application/6.3.69.8341/resultui/frame/javascript:void(0);) | Oral |
| Systemic Antihistamines | Systemic Antihistamines | [Azelastine](C:/Users/Administrator/AppData/Local/Youdao/Dict/Application/6.3.69.8341/resultui/frame/javascript:void(0);) | Oral |
| Systemic Antihistamines | Systemic Antihistamines | [Desloratadine](C:/Users/Administrator/AppData/Local/Youdao/Dict/Application/6.3.69.8341/resultui/frame/javascript:void(0);) | Oral |
| Systemic Antihistamines | Systemic Antihistamines | [Fexofenadine](C:/Users/Administrator/AppData/Local/Youdao/Dict/Application/6.3.69.8341/resultui/frame/javascript:void(0);) | Oral |
| Systemic Antihistamines | Systemic Antihistamines | Compound loratadine | Oral |
| Systemic Antihistamines | Systemic Antihistamines | Desloratadine Citrate Disodium | Oral |
| Systemic Antihistamines | Systemic Antihistamines | [Rupatadine](C:/Users/Administrator/AppData/Local/Youdao/Dict/Application/6.3.69.8341/resultui/frame/javascript:void(0);) | Oral |
| Systemic Antihistamines | Systemic Antihistamines | Loratadine | Oral |
| Systemic Antihistamines | Systemic Antihistamines | [Mizolastine](C:/Users/Administrator/AppData/Local/Youdao/Dict/Application/6.3.69.8341/resultui/frame/javascript:void(0);) | Oral |
| Systemic Antihistamines | Systemic Antihistamines | [Triprolidine](C:/Users/Administrator/AppData/Local/Youdao/Dict/Application/6.3.69.8341/resultui/frame/javascript:void(0);) | Oral |
| Systemic Antihistamines | Systemic Antihistamines | [Decloxizine](C:/Users/Administrator/AppData/Local/Youdao/Dict/Application/6.3.69.8341/resultui/frame/javascript:void(0);) | Oral |
| Systemic Antihistamines | Systemic Antihistamines | [Cyproheptadine](C:/Users/Administrator/AppData/Local/Youdao/Dict/Application/6.3.69.8341/resultui/frame/javascript:void(0);) | Oral |
| Systemic Antihistamines | Systemic Antihistamines | [Setastine](C:/Users/Administrator/AppData/Local/Youdao/Dict/Application/6.3.69.8341/resultui/frame/javascript:void(0);) | Oral |
| Systemic Antihistamines | Systemic Antihistamines | [Terfenadine](C:/Users/Administrator/AppData/Local/Youdao/Dict/Application/6.3.69.8341/resultui/frame/javascript:void(0);) | Oral |
| Systemic Antihistamines | Systemic Antihistamines | [Ebastine](C:/Users/Administrator/AppData/Local/Youdao/Dict/Application/6.3.69.8341/resultui/frame/javascript:void(0);) | Oral |
| Systemic Antihistamines | Systemic Antihistamines | [Diphenhydramine](C:/Users/Administrator/AppData/Local/Youdao/Dict/Application/6.3.69.8341/resultui/frame/javascript:void(0);) | Oral |
| Systemic Antihistamines | Systemic Antihistamines | Diphenhydramine menthol | Oral |
| Systemic Antihistamines | Systemic Antihistamines | Phenhydramine pseudoephedrine | Oral |
| Systemic Antihistamines | Systemic Antihistamines | [Dimenhydrinate](C:/Users/Administrator/AppData/Local/Youdao/Dict/Application/6.3.69.8341/resultui/frame/javascript:void(0);) | Oral |
| Systemic Antihistamines | Systemic Antihistamines | Acetaminophen isopropyl | [Injection](C:/Users/Administrator/AppData/Local/Youdao/Dict/Application/6.3.69.8341/resultui/frame/javascript:void(0);) |
| Systemic Antihistamines | Systemic Antihistamines | Dioxopromethazine | Oral |
| Systemic Antihistamines | Systemic Antihistamines | Compound diphenhydramine | Oral |
| Systemic Antihistamines | Systemic Antihistamines | [Chlorpheniramine](C:/Users/Administrator/AppData/Local/Youdao/Dict/Application/6.3.69.8341/resultui/frame/javascript:void(0);) | Oral |
| Systemic Antihistamines | Systemic Antihistamines | [Chlorcyclizine](C:/Users/Administrator/AppData/Local/Youdao/Dict/Application/6.3.69.8341/resultui/frame/javascript:void(0);) | Oral |
| Systemic Antihistamines | Systemic Antihistamines | [Clemastine](C:/Users/Administrator/AppData/Local/Youdao/Dict/Application/6.3.69.8341/resultui/frame/javascript:void(0);) | Oral |
| Systemic Antihistamines | Systemic Antihistamines | [tripelennamine](C:/Users/Administrator/AppData/Local/Youdao/Dict/Application/6.3.69.8341/resultui/frame/javascript:void(0);) | Oral |
| Systemic Antihistamines | Systemic Antihistamines | Norepinephrine brompheniramine | Oral |
| Systemic Antihistamines | Systemic Antihistamines | [Cyclizine](C:/Users/Administrator/AppData/Local/Youdao/Dict/Application/6.3.69.8341/resultui/frame/javascript:void(0);) | Oral |
| Systemic Antihistamines | Systemic Antihistamines | Dioxoprothazine | Oral |
| Systemic Antihistamines | Systemic Antihistamines | [Cetirizine](C:/Users/Administrator/AppData/Local/Youdao/Dict/Application/6.3.69.8341/resultui/frame/javascript:void(0);) | Oral |
| Systemic Antihistamines | Systemic Antihistamines | Cetirizine/pseudoephedrine | Oral |
| Systemic Antihistamines | Systemic Antihistamines | Promethazine | Oral |
| Systemic Antihistamines | Systemic Antihistamines | Promethazine | Oral |
| Systemic Antihistamines | Systemic Antihistamines | Promethazine Bile | Oral |
| Systemic Antihistamines | Systemic Antihistamines | Polyphenol pentamethazine | Oral |
| Systemic Antihistamines | Systemic Antihistamines | [Levocetirizine](C:/Users/Administrator/AppData/Local/Youdao/Dict/Application/6.3.69.8341/resultui/frame/javascript:void(0);) | Oral |
| Other anti-asthma and COPD drugs | Other anti-asthma and COPD drugs | [Ephedrine](C:/Users/Administrator/AppData/Local/Youdao/Dict/Application/6.3.69.8341/resultui/frame/javascript:void(0);) | Oral |
| Other anti-asthma and COPD drugs | Other anti-asthma and COPD drugs | [Ephedrine](C:/Users/Administrator/AppData/Local/Youdao/Dict/Application/6.3.69.8341/resultui/frame/javascript:void(0);) | [Injection](C:/Users/Administrator/AppData/Local/Youdao/Dict/Application/6.3.69.8341/resultui/frame/javascript:void(0);) |
| Other anti-asthma and COPD drugs | Other anti-asthma and COPD drugs | [Pranlukast](C:/Users/Administrator/AppData/Local/Youdao/Dict/Application/6.3.69.8341/resultui/frame/javascript:void(0);) | Oral |
| Other anti-asthma and COPD drugs | Other anti-asthma and COPD drugs | Compound ephedrine | Oral |
| Other anti-asthma and COPD drugs | Other anti-asthma and COPD drugs | [Methoxyphenamine](C:/Users/Administrator/AppData/Local/Youdao/Dict/Application/6.3.69.8341/resultui/frame/javascript:void(0);) | Oral |
| Other anti-asthma and COPD drugs | Other anti-asthma and COPD drugs | Compound atropine ephedrine | Oral |
| Other anti-asthma and COPD drugs | Other anti-asthma and COPD drugs | Compound atropine ephedrine | [Injection](C:/Users/Administrator/AppData/Local/Youdao/Dict/Application/6.3.69.8341/resultui/frame/javascript:void(0);) |
| Other anti-asthma and COPD drugs | Other anti-asthma and COPD drugs | Compound methoxamine | Oral |
| Other anti-asthma and COPD drugs | Other anti-asthma and COPD drugs | Compound methamphetamine hydrochloride | Oral |
| Other anti-asthma and COPD drugs | Other anti-asthma and COPD drugs | Compound polyphenols ephedrine | Oral |
| Other anti-asthma and COPD drugs | Other anti-asthma and COPD drugs | [Methylephedrine](C:/Users/Administrator/AppData/Local/Youdao/Dict/Application/6.3.69.8341/resultui/frame/javascript:void(0);) | Oral |
| Other anti-asthma and COPD drugs | Other anti-asthma and COPD drugs | Baicalin | Oral |
| Other anti-asthma and COPD drugs | Other anti-asthma and COPD drugs | Aminophylline Bromhexine and Chlorphenamine | Oral |
| Other anti-asthma and COPD drugs | Other anti-asthma and COPD drugs | Ephedrine phenhydramine | Oral |
| Other anti-asthma and COPD drugs | Other anti-asthma and COPD drugs | Cromolyn sodium | [Injection](C:/Users/Administrator/AppData/Local/Youdao/Dict/Application/6.3.69.8341/resultui/frame/javascript:void(0);) |
| Other anti-asthma and COPD drugs | Other anti-asthma and COPD drugs | Cromolyn sodium | Oral |
| Other anti-asthma and COPD drugs | Other anti-asthma and COPD drugs | Cromolyn sodium | [Injection](C:/Users/Administrator/AppData/Local/Youdao/Dict/Application/6.3.69.8341/resultui/frame/javascript:void(0);) |
| Other anti-asthma and COPD drugs | Other anti-asthma and COPD drugs | Asarone | [Injection](C:/Users/Administrator/AppData/Local/Youdao/Dict/Application/6.3.69.8341/resultui/frame/javascript:void(0);) |
| Other anti-asthma and COPD drugs | Other anti-asthma and COPD drugs | Sodium chloride | [Injection](C:/Users/Administrator/AppData/Local/Youdao/Dict/Application/6.3.69.8341/resultui/frame/javascript:void(0);) |
| Other anti-asthma and COPD drugs | Other anti-asthma and COPD drugs | Scopolamine bromide | [Inhalation](C:/Users/Administrator/AppData/Local/Youdao/Dict/Application/6.3.69.8341/resultui/frame/javascript:void(0);) |
