# Supplementary material for: Characteristics of medicine use for children with asthma in China: a nationwide population-based study
Source: BMC Pediatr. 2022 Dec 28;22:740. doi: 10.1186/s12887-022-03720-5 (PMC9795755; doi:10.1186/s12887-022-03720-5)
Supplement: Supplementary file 2 — Additional file 2. Supplementary Table 2. Therapeutic combination models. [file 12887_2022_3720_MOESM2_ESM.doc]

Supplementary Table 2. Therapeutic combination models

| Therapeutic combination models | | Numbers of people |
| --- | --- | --- |
| ICS | | 308 |
| ICS+LABA+SABA(Inhalation)+LTRA+SAAC | | 158 |
| LTRA | | 131 |
| ICS+SABA(Inhalation)+SAAC | | 120 |
| ICS+LABA+LTRA+SAAC | | 87 |
| ICS+SAAC | | 83 |
| ICS+LABA+OCS+SABA(Inhalation)+LTRA+SAAC | | 63 |
| ICS+LABA+SABA(Inhalation)+SAAC | | 61 |
| ICS+LTRA+SAAC | | 59 |
| LABA+LTRA | | 57 |
| ICS+LABA+SAAC | | 43 |
| ICS+OCS+SABA(Inhalation)+LTRA+SAAC | | 43 |
| OCS+Theophylline | | 43 |
| OCS | | 41 |
| LABA | | 29 |
| ICS/LABA | | 25 |
| ICS+OCS+SABA(Inhalation)+SAAC | | 24 |
| ICS+OCS+SABA(Oral)+Theophylline | | 24 |
| Theophylline | | 23 |
| ICS+LABA+OCS+LTRA+SAAC | | 22 |
| ICS+SABA(Inhalation) | | 19 |
| ICS+OCS+SAAC | | 19 |
| ICS/LABA+LTRA | | 16 |
| ICS+LTRA | | 16 |
| ICS+ICS/LABA+SABA(Inhalation)+LTRA+SAAC | | 15 |
| ICS | | 15 |
| ICS+LABA+OCS+SABA(Inhalation)+SAAC | | 15 |
| ICS+LABA+OCS+SAAC | | 14 |
| ICS+SABA(Inhalation)+LTRA | | 13 |
| OCS+LTRA | | 12 |
| ICS+LABA+OCS+SABA(Inhalation)+Theophylline+LTRA+SAAC | | 12 |
| ICS+OCS+Theophylline+LTRA+SAAC | | 12 |
| ICS+OCS+SABA(Inhalation)+Theophylline+LTRA+SAAC | | 12 |
| ICS+LABA+SABA(Inhalation)+SABA（Oral）+LTRA+SAAC | | 12 |
| OCS+SABA（Oral）+Theophylline | | 12 |
| ICS+LABA+OCS+SABA(Inhalation)+SABA（Oral）+LTRA+SAAC | | 11 |
| ICS+SABA(Inhalation)+SABA（Oral）+LTRA+SAAC | | 10 |
| ICS+LABA+ICS/LABA+OCS+SABA(Inhalation)+LTRA+SAAC | | 10 |
| ICS+OCS+SABA(Inhalation) | | 10 |
| ICS+LABA+ICS/LABA+LTRA+SAAC | | 9 |
| ICS+LABA+LTRA | | 9 |
| ICS+LABA+ICS/LABA+SABA(Inhalation)+LTRA+SAAC | | 9 |
| ICS+OCS+LTRA+SAAC | | 9 |
| ICS+LABA+SABA（Oral）+LTRA+SAAC | | 9 |
| ICS+SABA(Inhalation)+Theophylline | | 9 |
| LABA+ICS/LABA | | 8 |
| OCS+SABA(Inhalation)+Theophylline | | 8 |
| ICS+Theophylline+SAAC | | 7 |
| ICS+LABA+OCS+SABA(Inhalation)+LTRA | | 7 |
| ICS+SABA(Inhalation)+Theophylline+LTRA+SAAC | | 7 |
| ICS+SABA(Inhalation)+SABA（Oral）+LTRA | | 7 |
| ICS+SABA（Oral）+SAAC | | 7 |
| ICS+LABA+SABA(Inhalation)+LTRA | | 7 |
| ICS+OCS+SABA(Inhalation)+Theophylline | | 6 |
| ICS+OCS+Theophylline+SAAC | | 6 |
| SAAC | | 6 |
| ICS+SABA（Oral）+LTRA+SAAC | | 6 |
| ICS+OCS+SABA(Inhalation)+SABA（Oral）+Theophylline+SAAC | | 6 |
| SABA（Oral） | | 5 |
| ICS+OCS+SABA(Inhalation)+Theophylline+SAAC | | 5 |
| ICS+ICS/LABA+SABA(Inhalation)+SAAC | | 5 |
| ICS+OCS | | 5 |
| ICS+OCS+SABA(Inhalation)+SABA（Oral）+LTRA+SAAC | | 5 |
| LABA+OCS | | 5 |
| SABA(Inhalation)+LTRA | | 4 |
| ICS+LABA+OCS+Theophylline+LTRA+SAAC | | 4 |
| ICS+LABA+OCS+SABA（Oral）+Theophylline+LTRA+SAAC | | 4 |
| ICS+OCS+Theophylline | | 4 |
| OCS+SABA(Inhalation)+SAAC | | 4 |
| SABA(Inhalation) | | 4 |
| ICS+OCS+SABA(Inhalation)+LTRA | | 4 |
| ICS+OCS+SABA(Inhalation)+SABA（Oral）+Theophylline+LTRA+SAAC | | 4 |
| SABA(Inhalation)+LTRA+SAAC | | 4 |
| ICS+OCS+SABA(Inhalation)+Theophylline+LTRA | | 4 |
| LABA+OCS+LTRA | | 4 |
| OCS+Theophylline+SAAC | | 4 |
| ICS+LABA | | 4 |
| ICS+OCS+SABA(Inhalation)+SABA（Oral）+Theophylline | | 4 |
| ICS+OCS+SABA(Inhalation)+SABA（Oral）+Theophylline+LTRA | | 4 |
| ICS+LABA+SABA（Oral）+LTRA | | 3 |
| ICS+LABA+SABA(Inhalation)+Theophylline+SAAC | | 3 |
| ICS/LABA+OCS | | 3 |
| SABA（Oral）+LTRA | | 3 |
| ICS+LABA+SABA（Oral）+SAAC | | 3 |
| SABA(Inhalation)+SAAC | | 3 |
| ICS+OCS+SABA(Inhalation)+Theophylline+SAAC+Magnesium sulfate | | 3 |
| ICS+ICS/LABA+SABA(Inhalation)+LTRA | | 3 |
| LABA+ICS/LABA+LTRA | | 3 |
| ICS+LABA+OCS+SABA（Oral）+LTRA+SAAC | | 3 |
| LABA+OCS+SABA(Inhalation)+Theophylline+SAAC | | 3 |
| ICS+LABA+ICS/LABA+SAAC | | 3 |
| ICS+LABA+ICS/LABA+SABA(Inhalation)+SAAC | | 3 |
| ICS+OCS+SABA（Oral）+Theophylline+LTRA | | 3 |
| ICS+LABA+SABA(Inhalation)+Theophylline+LTRA+SAAC | | 3 |
| ICS+LABA+ICS/LABA+LTRA | | 3 |
| ICS+ICS/LABA+SABA(Inhalation)+SABA（Oral）+SAAC | | 3 |
| ICS+Theophylline+LTRA+SAAC | | 3 |
| ICS+ICS/LABA+LTRA | | 3 |
| ICS+LABA+OCS+SABA(Inhalation)+Theophylline+LTRA | | 3 |
| ICS+ICS/LABA+OCS+SABA(Inhalation)+LTRA+SAAC | | 3 |
| ICS+SABA(Inhalation)+Theophylline+LTRA | | 3 |
| ICS+SABA(Inhalation)+SABA（Oral）+SAAC | | 3 |
| ICS+LABA+SABA(Inhalation) | | 3 |
| ICS+ICS/LABA+LTRA+SAAC | | 3 |
| OCS+Theophylline+LTRA | | 3 |
| ICS+LABA+OCS+SABA(Inhalation) | | 3 |
| ICS+LABA+Theophylline+LTRA+SAAC | | 3 |
| ICS/LABA+SABA(Inhalation) | | 3 |
| OCS+SABA(Inhalation)+SABA（Oral）+Theophylline | | 3 |
| ICS+LABA+SABA(Inhalation)+Theophylline | | 2 |
| ICS+LABA+SABA(Inhalation)+SABA（Oral）+SAAC | | 2 |
| ICS+LABA+ICS/LABA+OCS+SAAC | | 2 |
| ICS/LABA+LTRA+SAAC | | 2 |
| ICS/LABA+OCS+SABA（Oral）+Theophylline+LTRA | | 2 |
| ICS/LABA+OCS+Theophylline | | 2 |
| ICS/LABA+Theophylline | | 2 |
| ICS+ICS/LABA+OCS+SABA(Inhalation)+SAAC | | 2 |
| ICS+ICS/LABA+SABA(Inhalation)+SABA（Oral）+LTRA | | 2 |
| ICS+ICS/LABA+SABA(Inhalation)+SABA（Oral）+LTRA+SAAC | | 2 |
| ICS+LABA+OCS+SABA(Inhalation)+SABA（Oral）+SAAC | | 2 |
| ICS+LABA+ICS/LABA+OCS+Theophylline+LTRA+SAAC | | 2 |
| ICS+LABA+OCS+Theophylline+SAAC | | 2 |
| ICS+LABA+ICS/LABA+SABA（Oral）+LTRA+SAAC | | 2 |
| ICS+LABA+OCS+LTRA | | 2 |
| ICS+LABA+OCS+SABA(Inhalation)+SABA（Oral）+LTRA | | 2 |
| ICS+LABA+OCS+SABA(Inhalation)+SABA（Oral）+Theophylline+LTRA+SAAC | | 2 |
| ICS+LABA+OCS+SABA(Inhalation)+SABA（Oral）+Theophylline+SAAC | | 2 |
| ICS+LABA+OCS+SABA(Inhalation)+Theophylline | | 2 |
| ICS+LABA+OCS+SABA(Inhalation)+Theophylline+SAAC | | 2 |
| ICS+LABA+ICS/LABA+OCS+LTRA+SAAC | | 2 |
| ICS+SABA(Inhalation)+SABA（Oral）+Theophylline+LTRA | | 2 |
| OCS+SABA(Inhalation)+Theophylline+SAAC | | 2 |
| LABA+OCS+SABA(Inhalation)+Theophylline+LTRA | | 2 |
| OCS+SABA（Oral）+Theophylline+LTRA | | 2 |
| LABA+OCS+Theophylline+LTRA | | 2 |
| LABA+OCS+Theophylline+SAAC | | 2 |
| OCS+SABA(Inhalation)+Theophylline+LTRA | | 2 |
| ICS+Theophylline+LTRA | | 2 |
| OCS+SABA（Oral）+SAAC | | 2 |
| ICS+SABA(Inhalation)+Theophylline+SAAC | | 2 |
| LABA+OCS+SABA(Inhalation)+Theophylline | | 2 |
| OCS+SAAC | | 2 |
| OCS+SABA（Oral）+Theophylline+SAAC | | 2 |
| ICS+OCS+SABA（Oral）+SAAC | | 2 |
| LTRA+SAAC | | 2 |
| Theophylline+LTRA | | 2 |
| ICS+OCS+LTRA | | 2 |
| ICS+OCS+SABA（Oral） | | 2 |
| LABA+SABA(Inhalation)+LTRA+SAAC | | 2 |
| ICS+OCS+SABA（Oral）+Theophylline+SAAC | | 2 |
| ICS+OCS+SABA(Inhalation)+SABA（Oral）+Theophylline+LTRA+SAAC+Magnesium sulfate | | 2 |
| ICS+OCS+SABA(Inhalation)+SABA（Oral）+SAAC | | 2 |
| ICS+ICS/LABA+SABA(Inhalation) | | 1 |
| OCS+SABA(Inhalation)+Theophylline+LTRA+SAAC | | 1 |
| ICS+ICS/LABA+Theophylline+SAAC | | 1 |
| ICS+ICS/LABA+SABA(Inhalation)+Theophylline+SAAC | | 1 |
| ICS+ICS/LABA+OCS+Theophylline+LTRA | | 1 |
| OCS+SABA（Oral）+Theophylline+SAAC+Magnesium sulfate | | 1 |
| OCS+SABA(Inhalation)+LTRA | | 1 |
| ICS+ICS/LABA+SABA（Oral）+Theophylline+LTRA+SAAC | | 1 |
| ICS+LABA+ICS/LABA | | 1 |
| OCS+Theophylline+LTRA+SAAC | | 1 |
| ICS+ICS/LABA+SAAC | | 1 |
| ICS+ICS/LABA+Theophylline+LTRA+SAAC | | 1 |
| OCS+SABA(Inhalation) | | 1 |
| OCS+SABA（Oral）+Theophylline+LTRA+Magnesium sulfate | | 1 |
| ICS+ICS/LABA+OCS+SAAC | | 1 |
| ICS/LABA+SABA(Inhalation)+LTRA | | 1 |
| Theophylline+SAAC | | 1 |
| SABA(Inhalation)+Theophylline+Magnesium sulfate | | 1 |
| SABA(Inhalation)+Theophylline+SAAC+Magnesium sulfate | | 1 |
| SABA（Oral）+SAAC | | 1 |
| ICS/LABA+OCS+LTRA | | 1 |
| ICS/LABA+OCS+SABA（Oral） | | 1 |
| ICS/LABA+OCS+SABA（Oral）+Theophylline | | 1 |
| SABA（Oral）+Theophylline | | 1 |
| ICS/LABA+OCS+SABA(Inhalation)+Theophylline+SAAC | | 1 |
| ICS+ICS/LABA+OCS+SABA（Oral）+LTRA | | 1 |
| OCS+Theophylline+Magnesium sulfate | | 1 |
| ICS+ICS/LABA+OCS+SABA(Inhalation)+Theophylline+LTRA+SAAC | | 1 |
| ICS/LABA+SABA(Inhalation)+SABA（Oral）+LTRA | | 1 |
| ICS/LABA+SABA(Inhalation)+SAAC | | 1 |
| OCS+Theophylline+SAAC+Magnesium sulfate | | 1 |
| ICS/LABA+SAAC | | 1 |
| ICS+ICS/LABA | | 1 |
| ICS+LABA+ICS/LABA+OCS+SABA(Inhalation)+Theophylline+LTRA+SAAC | | 1 |
| OCS+SABA(Inhalation)+SAAC+Magnesium sulfate | | 1 |
| ICS+ICS/LABA+OCS+SABA（Oral）+Theophylline+LTRA | | 1 |
| ICS+ICS/LABA+OCS+SABA（Oral）+Theophylline+SAAC | | 1 |
| ICS+ICS/LABA+OCS+SABA(Inhalation)+SABA（Oral）+Theophylline+LTRA | | 1 |
| ICS/LABA+SABA（Oral）+LTRA | | 1 |
| ICS+SABA（Oral）+Theophylline+LTRA | | 1 |
| ICS+LABA+ICS/LABA+OCS+SABA(Inhalation)+SABA（Oral）+Theophylline+LTRA+SAAC | | 1 |
| LABA+OCS+SABA（Oral）+Theophylline | | 1 |
| LABA+LTRA+SAAC | | 1 |
| ICS+LABA+OCS+SABA(Inhalation)+SABA（Oral）+Theophylline | | 1 |
| LABA+ICS/LABA+SABA(Inhalation)+LTRA | | 1 |
| ICS+LABA+OCS+SABA(Inhalation)+SABA（Oral）+Theophylline+LTRA+SAAC+Magnesium sulfate | | 1 |
| LABA+ICS/LABA+SABA(Inhalation) | | 1 |
| LABA+ICS/LABA+LTRA+SAAC | | 1 |
| ICS+Theophylline | | 1 |
| LABA+OCS+SABA（Oral）+Theophylline+SAAC | | 1 |
| ICS+LABA+OCS+SABA(Inhalation)+Theophylline+LTRA+Magnesium sulfate | | 1 |
| LABA+OCS+SABA（Oral）+SAAC+Magnesium sulfate | | 1 |
| ICS+SABA（Oral） | | 1 |
| ICS+LABA+OCS+Theophylline+LTRA+SAAC+Magnesium sulfate | | 1 |
| ICS+OCS+Theophylline+LTRA | | 1 |
| ICS+OCS+SABA(Inhalation)+Theophylline+LTRA+SAAC+Magnesium sulfate | | 1 |
| ICS+LABA+SABA（Oral） | | 1 |
| ICS+OCS+SABA(Inhalation)+SABA（Oral）+Theophylline+SAAC+Magnesium sulfate | | 1 |
| ICS+LABA+SABA（Oral）+Theophylline | | 1 |
| ICS+LABA+Theophylline+SAAC | | 1 |
| ICS+LABA+Theophylline | | 1 |
| ICS+LABA+OCS+SABA(Inhalation)+Theophylline+LTRA+SAAC+Magnesium sulfate | | 1 |
| ICS+LABA+ICS/LABA+SABA(Inhalation)+SABA（Oral）+LTRA+SAAC | | 1 |
| ICS+LABA+ICS/LABA+OCS+SABA（Oral）+LTRA+SAAC | | 1 |
| ICS+LABA+ICS/LABA+OCS+SABA（Oral）+Theophylline+LTRA+SAAC | | 1 |
| ICS+LABA+ICS/LABA+OCS+SABA(Inhalation) | | 1 |
| LABA+Theophylline+LTRA | | 1 |
| ICS+OCS+LTRA+SAAC+Magnesium sulfate | | 1 |
| ICS+LABA+ICS/LABA+OCS+SABA(Inhalation)+Theophylline | | 1 |
| ICS+LABA+SABA(Inhalation)+Theophylline+LTRA | | 1 |
| LABA+Theophylline | | 1 |
| LABA+SABA(Inhalation)+LTRA | | 1 |
| ICS+LABA+OCS+SABA(Inhalation)+LTRA+SAAC+Magnesium sulfate | | 1 |
| LABA+SABA（Oral）+Theophylline+LTRA | | 1 |
| OCS+SABA（Oral） | | 1 |
| LABA+OCS+Theophylline | | 1 |
| LABA+OCS+SABA(Inhalation)+Theophylline+Magnesium sulfate | | 1 |
| ICS+LABA+LTRA+SAAC+Magnesium sulfate | | 1 |
| LABA+OCS+SABA(Inhalation)+Theophylline+LTRA+Magnesium sulfate | | 1 |
| ICS+LABA+OCS+SABA（Oral）+LTRA | | 1 |
| LABA+OCS+SABA(Inhalation)+SABA（Oral）+Theophylline+Magnesium sulfate | | 1 |
| ICS+LABA+OCS+SABA（Oral）+Theophylline+SAAC | | 1 |
| ICS+LABA+OCS+SABA（Oral）+SAAC | | 1 |
| LABA+OCS+SABA(Inhalation) | | 1 |
| ICS+LABA+ICS/LABA+SABA(Inhalation)+LTRA | | 1 |
| Total Number | 238 | 2468 |

ICS: Inhaled Corticosteroids; LABA: Long-acting β_2_ Receptor Agonist; LTRA: Leukotriene Receptor Antagonist;

SABA: Short-acting β_2_ Receptor Agonist; SCS: Systemic Administration of Corticosteroids;

SAAC: Short-acting Anticholinergic Drugs.
